# Fosfomycin resistance mechanisms in *Enterobacterales*: an increasing threat

**DOI:** 10.3389/fcimb.2023.1178547

**Published:** 2023-07-04

**Authors:** Vittoria Mattioni Marchetti, Jaroslav Hrabak, Ibrahim Bitar

**Affiliations:** ^1^ Department of Microbiology, Faculty of Medicine, University Hospital in Pilsen, Charles University, Pilsen, Czechia; ^2^ Biomedical Center, Faculty of Medicine, Charles University, Pilsen, Czechia; ^3^ Unit of Microbiology and Clinical Microbiology, Department of Clinical-Surgical, Diagnostic and Pediatric Sciences, University of Pavia, Pavia, Italy

**Keywords:** fosfomycin, *Enterobacterales*, fosfomycin-resistance, fosfomycin-resistant determinant, epidemiology

## Abstract

Antimicrobial resistance is well-known to be a global health and development threat. Due to the decrease of effective antimicrobials, re-evaluation in clinical practice of old antibiotics, as fosfomycin (FOS), have been necessary. FOS is a phosphonic acid derivate that regained interest in clinical practice for the treatment of complicated infection by multi-drug resistant (MDR) bacteria. Globally, FOS resistant Gram-negative pathogens are raising, affecting the public health, and compromising the use of the antibiotic. In particular, the increased prevalence of FOS resistance (FOS^R^) profiles among *Enterobacterales* family is concerning. Decrease in FOS effectiveness can be caused by *i*) alteration of FOS influx inside bacterial cell or *ii*) acquiring antimicrobial resistance genes. In this review, we investigate the main components implicated in FOS flow and report specific mutations that affect FOS influx inside bacterial cell and, thus, its effectiveness. FosA enzymes were identified in 1980 from *Serratia marcescens* but only in recent years the scientific community has started studying their spread. We summarize the global epidemiology of FosA/C2/L1-2 enzymes among *Enterobacterales* family. To date, 11 different variants of FosA have been reported globally. Among acquired mechanisms, FosA3 is the most spread variant in *Enterobacterales*, followed by FosA7 and FosA5. Based on recently published studies, we clarify and represent the molecular and genetic composition of *fosA/C2* genes enviroment, analyzing the mechanisms by which such genes are slowly transmitting in emerging and high-risk clones, such as *E. coli* ST69 and ST131, and *K. pneumoniae* ST11. FOS is indicated as first line option against uncomplicated urinary tract infections and shows remarkable qualities in combination with other antibiotics. A rapid and accurate identification of FOS^R^ type in *Enterobacterales* is difficult to achieve due to the lack of commercial phenotypic susceptibility tests and of rapid systems for MIC detection.

## Highlights

*Antimicrobial resistance currently represents a concern for human health and the reintroduction in clinical practice of old antibiotics as fosfomycin can provide further option in treatment of multi-drug resistant (MDR) bacterial infections.
*However, there is a global increasement of fosfomycin resistance bacteria, especially *Enterobacterales*, reducing its effectiveness.
*Considering this increasement, it would be crucial to understand and clarify the several mechanisms involved in fosfomycin resistance among clinically and veterinary relevant *Enterobacterales*.
*Moreover, knowledge on the global epidemiology of acquired fosfomycin resistance genes would provide information about the major transmission routes of such resistance profiles.

## Introduction

Antimicrobial resistance (AMR) is one of the major global public health threats in 21^st^ century that affects prevention and treatment of a wide range of bacterial infections (Prestinaci et al., 2015). In the last 20 years, several strategies have been developed and suggested to combat AMR. In 2012, World Health Organization (WHO) published *The Evolving Threat of Antimicrobial Resistance – Options for Action*, which presented interventions that will strength the health systems and enhance surveillance through improving the usage of antimicrobials in hospitals and communities, infection prevention, and encouraging the development of appropriate new drugs and vaccines (Prestinaci et al., 2015). In accordance with WHO report published in 2020, 43 antibiotics and combinations are currently in clinical development and, since 2017, 11 new antimicrobial drugs have been approved for clinical use. However, WHO claims that none of the 43 antibiotics sufficiently address the problem of AMR in the most clinically problematic bacteria (e.g., *Escherichia coli*, *Klebsiella pneumoniae*). As the antibiotics availability is decreasing with time, the old antibiotics retaining effectiveness against some multi-drug resistant (MDR) pathogens are re-introduced (Theuretzbacher and Paul, 2015). This temporary solution allowed the renaissance of molecules such as colistin, nitrofurantoin and fosfomycin (FOS).

## Fosfomycin

**Figure 1:**
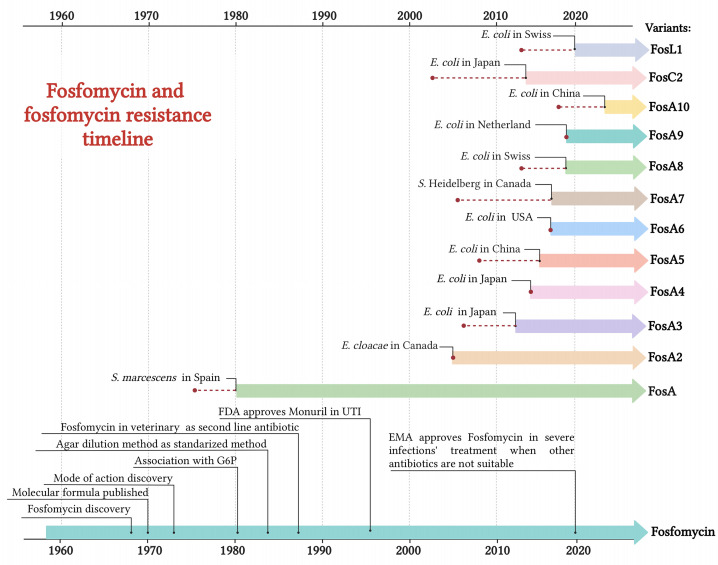
Timeline of FOS usage and the emergence of acquired FOS resistance determinants. Red dot = year of isolation. Created with BioRender.com.

FOS, originally called phosphonomycin, is a phosphonic acid derivate discovered in 1969 by the Medina Foundation (Fundación Medina, Granada, Spain) from soil *Streptomyces fradiae* and *Pseudomonas syringae.* The same year, Christensen et al. determined the FOS molecular formula (–)-(1R, 2S)-1,2-epoxy propyl phosphonic acid (Christensen et al., 1969). FOS interferes with the early stages of peptidoglycan production, inhibiting UDP-N-acetylglucosamine enolpyruvyl transferase (MurA) enzyme. MurA enzyme catalyzes the formation of peptidoglycan precursor, N-acetylmuramic acid. The binding of FOS to MurA and, thus, the inability to proceed in peptidoglycan formation result in a bactericidal activity of the drug (Candel et al., 2019). Since both Gram-positive and -negative bacteria requires the formation of N-acetylmuramic acid for peptidoglycan, FOS presents a broad-spectrum antibiotic activity against the main genera in clinical practice, including carbapenemase- and/or extended-spectrum β-lactamase (ESβL)-producing *Enterobacterales*, methicillin-resistant *Staphylococcus aureus* (MRSA), glycopeptide-resistant enterococci and multidrug-resistant (MDR) *Pseudomonas aeruginosa* (Putensen et al., 2019). Chemically, FOS has a simple structure consisting in an active epoxic group bonded, through a carbon molecule, to a phosphorous (Baron et al., 1986). FOS has some unique features such as low molecular weight (138.06 g/mol) and protein binding capabilities, providing it with high tissue penetration (volume of distribution of 0.3 L/kg) (Candel et al., 2019). FOS mode of action was first described in 1974 by Kahan and colleagues, and the *in vitro* standardization testing was provided by Andrews et al. in 1983 (Hirschl et al., 1980; Andrews et al., 1983). Despite FOS advantages, intravenous use of FOS almost disappeared from clinical practice, partly due to its incongruency of *in vitro* results in early susceptibility testing (Barnett et al., 1969). FOS is available in three formulations: two orally used calcium salt form (C_3_H_5_O_4_PCa;194.2) and FOS tromethamione (C_7_H_18_NO_7_P; 259.194), and an intravenously used disodium salt (C_3_H_5_O_4_PNa_2_; 182.03) (Falagas et al., 2016). In 1996, Food and Drugs Administration (FDA) approved the clinical use of oral FOS (Monurol) in the treatment of uncomplicated lower urinary tract infections (UTIs), as acute cystitis. In the following years, FOS oral formulation was also approved in perioperative prophylaxis for transrectal prostate biopsy in adult man, post-operative treatment of UTIs, recurrent UTIs, acute uncomplicated UTIs in children and acute cystitis during pregnancy. In 2020, the European Medicine Agency (EMA) approved FOS for infusion in the treatment of a wide range of conditions (e.g. complicated urinary tract infections, bone and joint infections, bacterial meningitis) when the commonly recommended drugs are considered inappropriate (**Figure 1**). Some European countries such as Austria, France, Germany, Greece, and Spain allow the use of FOS intravenously with other antibiotics, such as β-lactam antibiotics or fluoroquinolones in critically ill patients suffering from carbapenem-resistant Enterobacterales infections (Michalopoulos et al., 2011). This is due to FOS’ unique mechanism of action and to the absence of side effects as nephrotoxicity, typical of aminoglycosides or colistin (Michalopoulos et al., 2010). FOS usage in veterinary settings is forbidden in China and European countries, while in Central and South America regions, such as Brazil and Argentina, is largely administered in diseased broiler chickens and pigs (Pérez et al., 2014; Wang et al., 2017). In 2016, WHO categorized phosphonic acid derivatives as critically important antibiotic in human medicine highlighting their high frequency use in human medicine and their role as available therapy to treat serious bacterial infections in people. Despite the relevance in human medicine, data concerning FOS susceptibility profiles have not been included yet in annual report on antimicrobial resistance by WHO or ECDC. Consequently, the global epidemiology of FOS resistant profiles and FOS-modifying enzymes is still incomplete and not well monitored.

## FOS target

**Figure 2:**
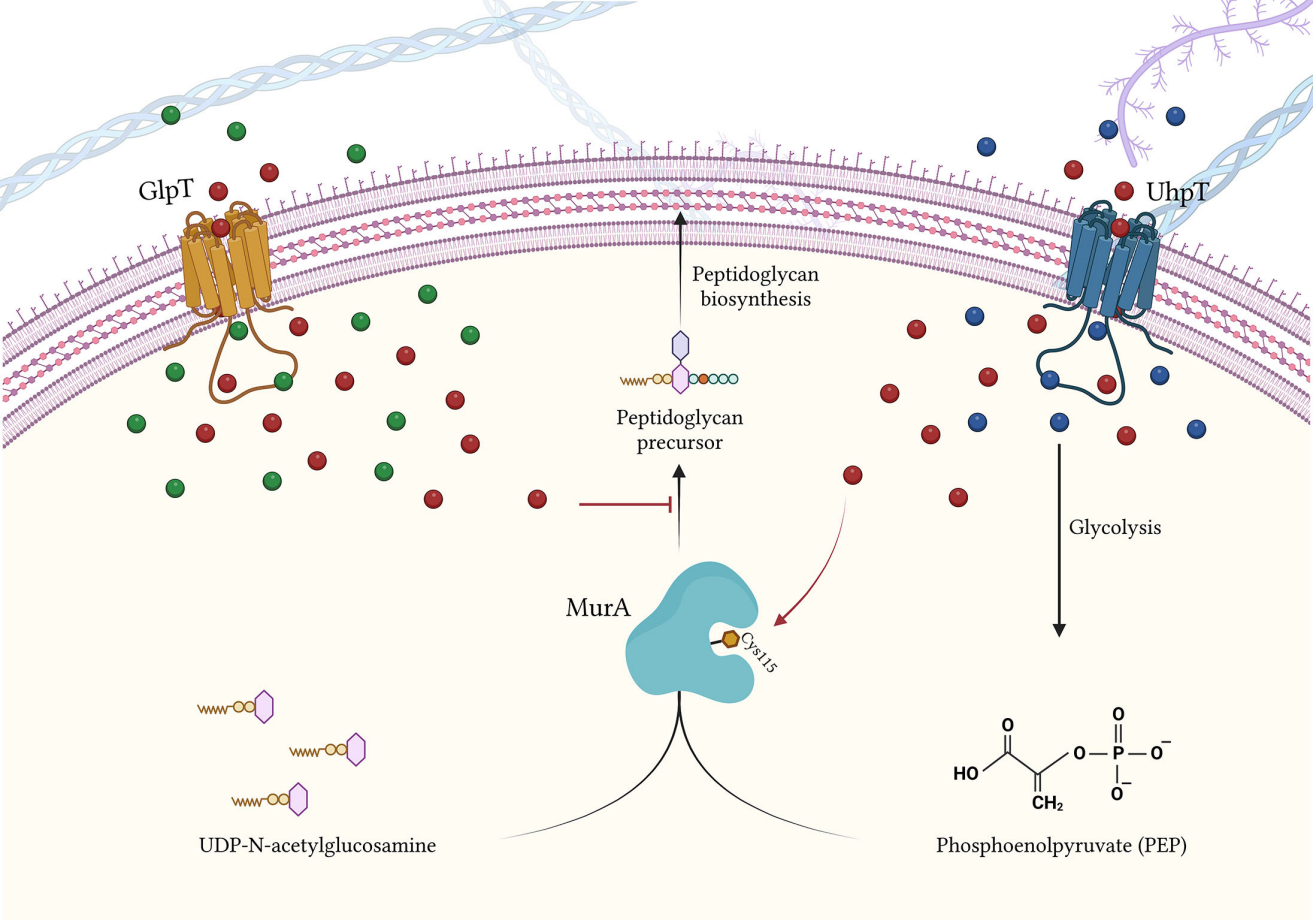
FOS influx inside the bacterial cell *via* GlpT and UhpT transportes, and FOS mode of action. Blue bubbles = G6P; green bubbles = G3P; red bubbles = FOS. Created with BioRender.com.

FOS binds and inhibits the UDP-GlcNAc enolpyruvyl transferase (MurA), acting as a phosphoenolpyruvate (PEP) analogue (Brown et al., 1995; Aghamali et al., 2019). MurA is a fundamental enzyme involved in the initial steps of peptidoglycan biosynthesis (Brown et al., 1995; Aghamali et al., 2019). FOS carries out its inhibiting activity to MurA through a covalent binding between the thyol group of a cysteine and the MurA active site, Cys115 (**Figure 2**). This inhibitory effect occurs in the cytoplasm and impairs an earlier stage of peptidoglycan biosynthesis when compared with that of β-lactamases or glycopeptides (Kahan et al., 1974; Eschenburg et al., 2005)..

## FOS transportation into the bacterial cell

The FOS intake has been mainly characterized in *E. coli*. To overcome bacterial wall, FOS takes advantage of the transportation activity of GlpT (glycerol-3-phosphate transporter) and UhpT (hexose-6-phosphate: phosphate antiporter) (Ambudkar et al., 1990; Aghamali et al., 2019) (**Figure 2**).

### GlpT transporter

**Figure 3:**
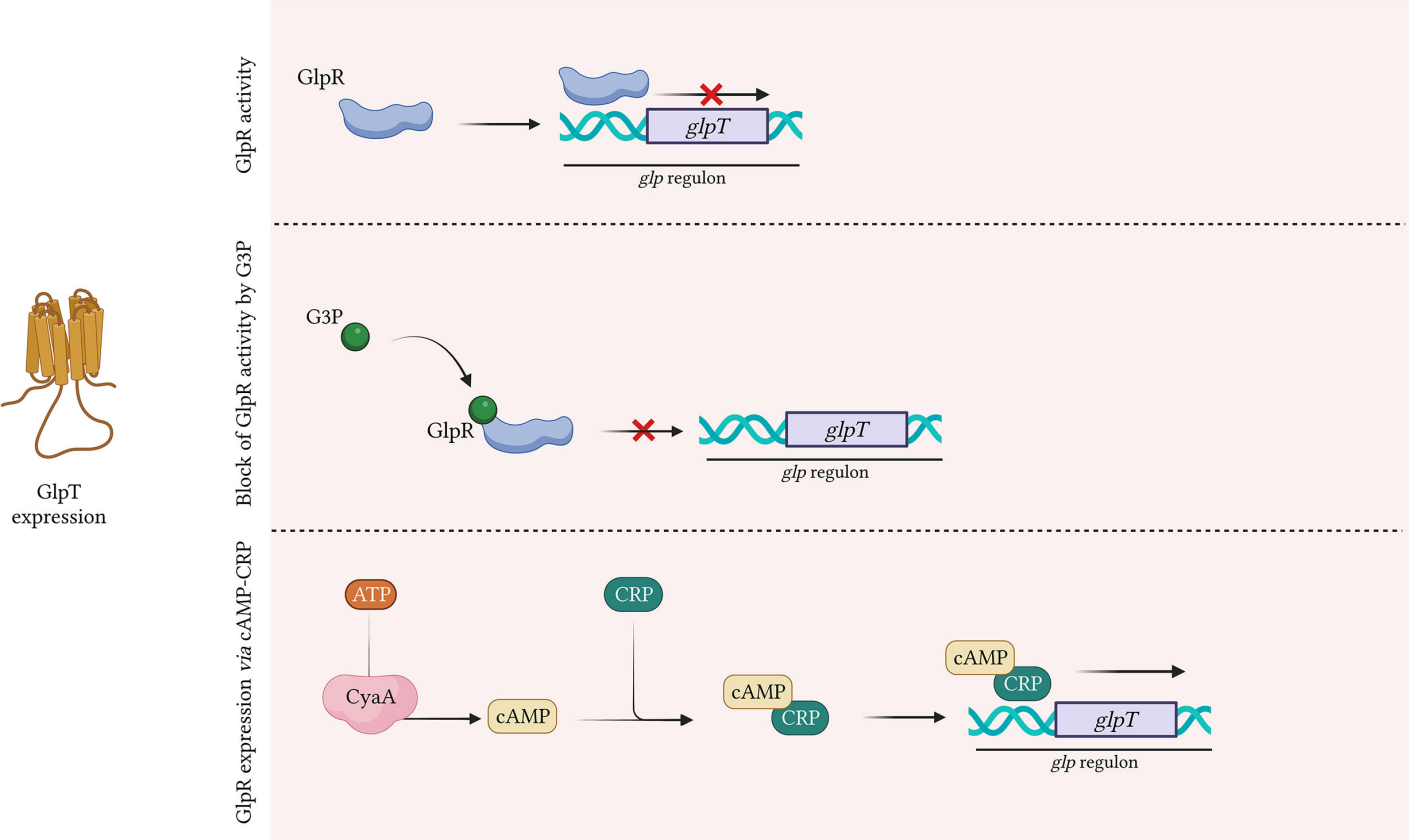
GlpT expression by GlpR. G3P positively regulates *glpT* expression. G3P binds GlpR repressor, reducing its affinity to *glpT* promotor. Without G3P, the repressor GlpR binds *glpT* promotor, derepressing glpT expression. CRP-bound cAMP binds *glpT* promotor and positively regulates *glpT* expression. Created with BioRender.com.

GlpT is a member of the organophosphate phosphate antiporter (OPA) family and is highly conserved in several species such as *Escherichia* spp., *Klebsiella* spp., *Salmonella* spp., and *Citrobacter* spp (Kahan et al., 1974).. GlpT is structured into two transmembrane domains, each composed of six highly conserved α-helices, that are linked by a long central loop (Lemieux et al., 2004). The *glpT* gene is part of the *glp* regulon, that controls the catabolism of G3P, glycerol and glycerophosphodiesters (Yang and Larson, 1998) (**Figure 3**). The extracellular G3P enters the bacterial cell through GlpT and control the expression of GlpT itself (Castañeda-García et al., 2013). In details, G3P binds to GlpR (G3P regulon repressor) that regulates the transcription of *glp* regulon, including *glpT* (Yang and Larson, 1998; Lemieux et al., 2004; Escapa et al., 2013) (**Figure 3**). In absence of G3P, GlpR binds to the operators of *glp* regulon, located in proximity of the promotor regions, and decreases the expression levels of *glp* regulon, including *glpT* (Yang and Larson, 1998) (**Figure 3**). When present, G3P binds to GlpR and lower GlpR-binding affinity with *glp* regulon, preventing the binding of GlpR to *glpT* promotor. The inability to bind the operator blocks *glpT* repression, leading to an increase of its expression levels (Cozzarelli et al., 1968; Law et al., 2009) (**Figure 3**).

### UhpT transporter

An alternative route for FOS influx is *via* UhpT transport system. UhpT is a monomer consisting of twelve transmembrane α-helical segments, which show high amino acid sequence homology with GlpT (Ambudkar et al., 1990). UhpT is a member of the Major Facilitator Superfamily (MFS) and promotes the entry of G6P, fructose-6-phosphate and mannose-6-phosphate inside bacterial cell (Hall and Maloney, 2005). The UhpT system is exclusive to *Enterobacteriaceae*, except for *Proteus* spp. and *Staphylococcus* spp (Silver, 2017)..

In the presence of G6P, UhpT expression is highly induced (Yang et al, 2016), leading to an increase of FOS flow inside the cell (Xu et al., 2017).

### UhpABC system

**Figure 4:**
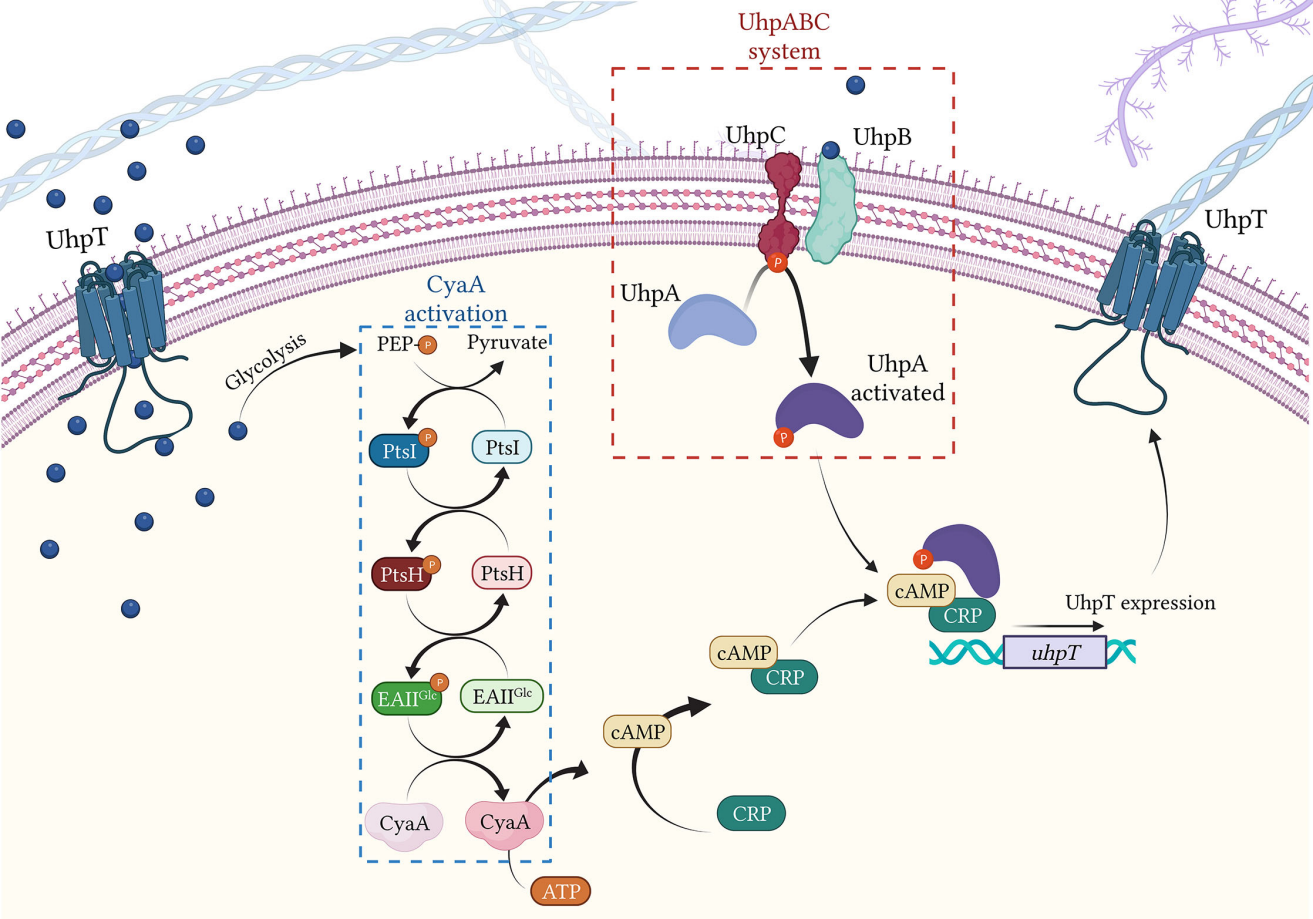
Regulation of UhpT expression by CyaA activation and UhpABC system. Blue bubbles = G6P; green bubbles = G3P; red bubbles = FOS. Created with BioRender.com.

To induce the expression of UhpT, G6P interacts with the UhpABC system, composed of three proteins: the transcriptional regulatory protein UhpA, the signal transduction histidine-protein kinase/phosphatase UhpB and the membrane sensor protein UhpC (Västermark and Saier, 2014) (**Figure 4**). UhpC senses external G6P and interacts with UhpB, stimulating the autokinase activity of UhpB (Friedrich and Kadner, 1987). The activated UhpB transfers its phosphate to UhpA, activating it. Thus, UhpA induces the transcription of *uhpT* by binding specifically to the *uhpT* promoter gene (Dahl et al., 1997) (**Figure 4**). In addition, to completely activate *uhpT* transcription, UhpA requires the presence of cAMP-CRP complex (Escapa et al., 2013).

### cAMP and adenylate cyclase CyaA

The transcription of both *glpT* and *uhpT* is under the control of the adenylate cyclase CyaA. CyaA catalyzes the formation of cAMP (cyclic adenosine monophosphate) from ATP (**Figure 4**). Once produced, cAMP showed high affinity with the transcriptional regulator CRP (DNA-binding transcriptional dual regulator) and binds together leading to the formation of the cAMP-CRP complex. Concerning UhpT expression, the cAMP-CRP complex binds the activated UhpA and together attach the *uhpT* promotor, inducing its transcription (Castañeda-García et al., 2013). Similarly, regarding GlpT expression, the cAMP-CRP complex alone attaches to *glpT* promotor (Castañeda-García et al., 2013) (**Figure 4**).

#### Activation of CyaA

The activation of CyaA requires the presence of G6P and of the PTS system, the carbohydrate phosphotransferase system (Postma et al., 1993) (**Figure 4**). The PTS system is a sugar-phosphorylating system described in *E. coli* and requires three different entities: Enzyme I (PtsI), the heat-stable, histidine-phosphorylatable protein HPr (PtsH) and Enzyme II (composed by the domains EIIA^Glc^) (Deutscher et al., 2014). Once in the bacterial cell, G6P enters the glycolysis cycle, which leads to the production of the PEP. The formed PEP undergoes to the PTS system, transferring a P group to PtsI (Deutscher et al., 2014). Thus, PtsI activates through phosphorylation PtsH, which consequently activates EIIA^Glc^, transferring the P group to EIIA^Glc^ (Saffen et al., 1987). Then, the activated EIIA^Glc^ induces the activation of CyaA (Mazé et al., 2014) (**Figure 4**).

#### Mechanisms of fosfomycin resistance

The recent use of FOS and co-selection phenomena have contributed to the development of FOS resistance and its dissemination. FOS^R^ mechanisms can be divided into three major groups: (a) modification of the antibiotic target MurA, (b) reduced permeability to FOS, and (c) acquisition of AMR genes. According to the recent literature, the reduction of FOS permeability is considered as the most frequent resistance mechanism (Nilsson et al., 2003; Castañeda-García et al., 2013; Silver, 2017).

### Modification of the target

FOS inactivates MurA by binding to its active site, Cys115 (Skarzynski et al., 1996). Kim and colleagues demonstrated that Cys115 substitutions in MurA, as Cys115Asp, lead to *in vitro* FOS^R^ (MIC > 512 mg/ml) in *E. coli* (Kim et al., 1996). However, mutations in MurA are uncommon in clinical isolates and none occurred in the catalytic site of MurA (Castañeda-García et al., 2013). Indeed, the first reports of mutations occurring in MurA from clinical *E. coli* isolates dated to 2010 in Japan, where the substitutions Asp369Asn and Leu370Ile were suggested to lead to development of FOS^R^ *in vivo* (Takahata et al., 2010). Both mutations, occurring in two highly conserved residues, decreasing the susceptibility to FOS with MIC up to 512 mg/ml (Takahata et al., 2010). Subsequently, mutations in MurA associated to FOS^R^ profiles have been detected from clinical *E. coli* isolates in China (Li et al., 2015; Bi et al., 2017), Taiwan (Tseng et al., 2015) and South Korea (Seok et al., 2020) (**Table 1**). Regarding clinical isolates of *K. pneumoniae*, Lu and coauthors reported several alterations in MurA sequence of ESβL-producing *K. pneumoniae* from Taiwan associated with FOS^R^ profiles (MICs = 128 μg/mL) (Lu et al., 2016). The exposition of FOS to bacterial strains can induce covalent modifications in MurA, increasing the enzyme synthesis (Marquardt et al., 1992). Interestingly, the overexpression of *murA* gene in *E. coli* is able to confer clinical levels of FOS^R^ (MIC=32 µg/mL) with a low fitness cost (5%) (Horii et al., 1999; Couce et al., 2012) (**Table 2**).

**Table 1:** Mutations impairing FOS resistance profiles in *E. coli*.

Enzyme	Mutations	Domain	Effect	Associated FOS MIC	Reference
**MurA**	No peptide	NA	Loss of function	512 μg/mL	(Bi et al., 2017)
	Ile28Asn	NA	Alteration of function	>256 μg/mL	(Bi et al., 2017)
	Phe30Leu	NA	Alteration of function	256 μg/mL	(Bi et al., 2017)
	Gln59Lys	NA	Alteration of function	256 μg/mL	(Li et al., 2015)
	Asn67Ile	NA	Alteration of function	256 μg/mL	(Tseng et al., 2015)
	Val146Ala	NA	Alteration of function	256 μg/mL	(Tseng et al., 2015)
	Phe151Ser	NA	Alteration of function	512 μg/mL	(Tseng et al., 2015)
	Ala154Thr	NA	Alteration of function	NA	(Seok et al., 2020)
	His159Tyr	NA	Alteration of function	256 μg/mL	(Tseng et al., 2015)
	Pro99Ser	NA	Alteration of function	NA	(Seok et al., 2020)
	Cys115Asp	Catalytic domain	Alteration of function	NA	(Kim et al., 1996)
	Cys115Glu	Catalytic domain	Loss of function	NA	(Kim et al., 1996)
	Glu139Lys	NA	Alteration of function	128 μg/mL	(Li et al., 2015)
	Trp164Ser	NA	Alteration of function	256 μg/mL	(Tseng et al., 2015)
	Asp369Asn	NA	MurA overexpression	512 μg/mL	(Takahata et al, 2010)
	Leu370Ile	NA	MurA overexpression	256 μg/mL	(Takahata et al, 2010)
	Val389Ile	NA	Alteration of function	128 μg/mL	(Li et al., 2015)
	Asp390Ala	NA	Alteration of function	128 μg/mL	(Li et al., 2015)
**GlpT**	Ile4Val	Cytoplasmic domain	NA	256 μg/mL	(Li et al., 2015)
	Ala16Thr	Cytoplasmic domain	Reducted permeability	NA	(Seok et al., 2020; Sorlozano-Puerto et al., 2020)
	W28del	Cytoplasmic domain	Loss of function	>128 μg/mL	(Mattioni Marchetti et al., 2023)
	Gly33Arg	Cytoplasmic domain	Loss of function	NA	(Takahata et al, 2010)
	Arg50Cys	Transmembrane	Reducted permeability	128 μg/mL	(Tseng et al., 2015)
	Met52Leu	Transmembrane	Reducted permeability	NA	(Sorlozano-Puerto et al., 2020)
	Gly84Asp	Transmembrane	Reducted permeability	NA	(Sorlozano-Puerto et al., 2020)
	Phe133Cys	Transmembrane	Reducted permeability	NA	(Sorlozano-Puerto et al., 2020)
	Gly135Trp	Transmembrane	Reducted permeability	NA	(Sorlozano-Puerto et al., 2020)
	Met136Lys	Transmembrane	Reducted permeability	NA	(Seok et al., 2020)
	Insertion Asp-Gly139	Transmembrane	Reducted permeability	NA	(Seok et al., 2020)
	Gly142Cys	Cytoplasmic domain	Reducted permeability	128 μg/mL	(Tseng et al., 2015)
	Thr144Pro	Cytoplasmic domain	Reducted permeability	256 μg/mL	43
	Val149Met	Cytoplasmic domain	Reducted permeability	128 μg/mL	(Tseng et al., 2015)
	Ala156Val	Cytoplasmic domain	Reducted permeability	128 μg/mL	(Tseng et al., 2015)
	Gly168Arg	Transmembrane	Reducted permeability	NA	(Seok et al., 2020)
	Ala197Val	Transmembrane	Reducted permeability	NA	(Sorlozano-Puerto et al., 2020)
	Pro173Ser	Transmembrane	Reducted permeability	256 μg/mL	(Li et al., 2015)
	Leu174Val	Transmembrane	Reducted permeability	512 μg/mL	(Tseng et al., 2015)
	Arg209His	Cytoplasmic domain	Reducted permeability	256 μg/mL	(Tseng et al., 2015)
	Asp220Asn	Cytoplasmic domain	Reducted permeability	NA	(Seok et al., 2020)
	Pro212Leu	Cytoplasmic domain	Reducted permeability	NA	(Sorlozano-Puerto et al., 2020)
	Leu373Arg	Transmembrane	Reducted permeability	NA	(Sorlozano-Puerto et al., 2020)
	Truncation to 206aa	NA	Loss of function	128 μg/mL	(Li et al., 2015)
	Gly437Cys	Cytoplasmic domain	Reducted permeability	>256 μg/mL	(Li et al., 2015)
	Glu448Lys	Cytoplasmic domain	Loss of function	NA	(Takahata et al, 2010)
	Leu297Phe	Transmembrane	Reducted permeability	NA	(Bi et al., 2017; Sorlozano-Puerto et al., 2020)
	Glu443Gln	Cytoplasmic domain	Reducted permeability	NA	(Sorlozano-Puerto et al., 2020)
	Gln444Glu	Cytoplasmic domain	Reducted permeability	NA	(Sorlozano-Puerto et al., 2020)
	Gly302Asp	Transmembrane	Reducted permeability	NA	(Sorlozano-Puerto et al., 2020)
	Phe176Leu	Transmembrane	NA	NA	(Ohkoshi et al., 2017)
	Phe176Ser	Transmembrane	Reducted permeability	128 μg/mL	(Tseng et al., 2015)
	Ile171Thr	Transmembrane	Reduced functionality	32 μg/mL	(Ohkoshi et al., 2017)
	Deletion aa155-158	Cytoplasmic domain	Loss of function	128 μg/mL	(Ohkoshi et al., 2017)
**UhpT**	Val18Leu	Cytoplasmic domain	Reducted permeability	128 μg/mL	(Tseng et al., 2015)
	Ser26Arg	Transmembrane	Reducted permeability	128 μg/mL	(Tseng et al., 2015)
	Trp44Cys	Transmembrane	Reducted permeability	128 μg/mL	(Tseng et al., 2015)
	Tyr60Phe	Periplasmic domain	Reducted permeability	NA	(Seok et al., 2020)
	Val85Leu	Cytoplasmic domain	Reducted permeability	256 μg/mL	(Tseng et al., 2015)
	Lys132Glu	Transmembrane	Reducted permeability	128 μg/mL	(Tseng et al., 2015)
	Gly134Asp	Transmembrane	Reducted permeability	128 μg/mL	(Tseng et al., 2015)
	Ile149Met	Cytoplasmic domain	Reducted permeability	128 μg/mL	(Tseng et al., 2015)
	Tyr165His	Transmembrane	Reducted permeability	128 μg/mL	(Tseng et al., 2015)
	No peptide	NA	Loss of function	>128 μg/mL	(Takahata et al, 2010; Li et al., 2015; Seok et al., 2020)
	Glu350Gln	Periplasmic domain	Reducted permeability	NA	(Takahata et al, 2010)
	ΔUhpT	NA	Loss of function	>64 μg/mL	(Ortiz-Padilla et al., 2022)
	Gln345Stop	Transmembrane	Loss of function	>1,024 μg/mL	(Ballestero-Téllez et al., 2017)
**UhpA**	Deletion 163-188aa	NA	Loss of function	1024 μg/mL	(Ohkoshi et al., 2017)
	Thr3Ala	Response regulatory domain	Reduced functionality	NA	(Ohkoshi et al., 2017)
	Met1Ile	NA	Reduced functionality	16 μg/mL	(Ohkoshi et al., 2017)
	Loss entire gene	NA	Loss of function	>32 μg/mL	(Ohkoshi et al., 2017)
	Truncation 283bp-591bp	NA	Premature stop codon	>1,024 μg/mL	(Lucas et al., 2017)
	Loss UhpT-UhpA-UhpC	NA	Loss of function	64 μg/mL	(Lucas et al., 2017)
**UhpB**	Gly469Arg	Cytoplasmic domain	Loss of function	128 μg/mL	(Cattoir et al., 2020)
	Thr27Stop	Transmembrane	Loss of function	>1,024 μg/mL	(Ballestero-Téllez et al., 2017)
	Gln262Stop	Transmembrane	Loss of function	>1,024 μg/mL	(Ballestero-Téllez et al., 2017)
	Trp181Stop	NA	Loss of function	>1,024 μg/mL	(Ballestero-Téllez et al., 2017)
	Leu255Stop	Transmembrane	Loss of function	>1,024 μg/mL	(Ballestero-Téllez et al., 2017)
**UhpC**	Phe384Leu	Transmembrane	Loss of function	128 μg/mL	(Cattoir et al., 2020)
	Thr72Pro	Transmembrane	NA	NA	(Ballestero-Téllez et al., 2017)
**CyaA**	His716Leu	Regulatory region	Reduced functionality	8 / 64 μg/mL	(Ohkoshi et al., 2017)
	Ser142Asn	Catalytic region	Reduced functionality	32 μg/mL	(Ohkoshi et al., 2017)
	Gly222Ser	Catalytic region	NA	NA	(Sorlozano-Puerto et al., 2020)
	Ser356Leu	Catalytic region	NA	NA	(Sorlozano-Puerto et al., 2020)
	Gly359Glu	Catalytic region	NA	NA	(Sorlozano-Puerto et al., 2020)

NA, Not Available.

**Table 2:** Mutations imparing FOS resistance profiles in *K. pneumoniae*.

Enzyme	Mutations	Domain	Effect	Associated FOS MIC	Reference
**MurA**	Gly118Asp	NA	Alteration of the target	128 μg/mL	(Seok et al., 2020)
	Glu130Lys	NA	Alteration of the target	128 μg/mL	(Lu et al., 2016)
	Thr214Ile	NA	Alteration of the target	256 μg/mL	(Lu et al., 2016)
	Asp259Asn	NA	Alteration of the target	128 μg/mL	(Lu et al., 2016)
	Asp260Tyr	NA	Alteration of the target	512 μg/mL	(Lu et al., 2016)
	Arg267Leu	NA	Alteration of the target	128 μg/mL	(Lu et al., 2016)
	Leu282Phe	NA	Alteration of the target	128 μg/mL	(Lu et al., 2016)
	Thr287Asn	NA	Alteration of the target	>256 μg/mL	(Lu et al., 2016)
	Thr307Lys	NA	Alteration of the target	>256 μg/mL	(Lu et al., 2016)
**GlpT**	Arg177Lys	Transmembrane	Reducted permeability	128 μg/mL	(Lu et al., 2016)
	Phe183Leu	Transmembrane	Reducted permeability	128 μg/mL	(Lu et al., 2016)
	Phe184Ile	NA	Reducted permeability	128 μg/mL	(Lu et al., 2016)
	Ser205Thr	Transmembrane	Reducted permeability	256 μg/mL	(Lu et al., 2016)
	Arg206Lys	Transmembrane	Reducted permeability	256 μg/mL	(Lu et al., 2016)
	Thr208Ser	Transmembrane	Reducted permeability	256 μg/mL	(Lu et al., 2016)
	Asp214Glu	NA	Reducted permeability	128 μg/mL	(Lu et al., 2016)
	Cys221Arg	NA	Reducted permeability	256 μg/mL	(Lu et al., 2016)
	Ile226Thr	NA	Reducted permeability	128 μg/mL	(Lu et al., 2016)
	Glu241Lys	NA	Reducted permeability	256 μg/mL	(Lu et al., 2016)
	Ala255Glu	Transmembrane	Reducted permeability	128 μg/mL	(Lu et al., 2016)
	Pro257Arg	Transmembrane	Reducted permeability	128 μg/mL	(Lu et al., 2016)
	Ile266Ser	Transmembrane	Reducted permeability	>128 μg/mL	(Lu et al., 2016)
	Asp274Val	NA	Reducted permeability	>128 μg/mL	(Wang et al., 2022)
	Asn278Lys	NA	Reducted permeability	128 μg/mL	(Lu et al., 2016)
	Ser283Cys	NA	Reducted permeability	512 μg/mL	(Lu et al., 2016)
	Ile293Phe	Transmembrane	Reducted permeability	512 μg/mL	(Lu et al., 2016)
	Glu299Asp	Transmembrane	Reducted permeability	>128 μg/mL	(Wang et al., 2022)
	Gly300Arg	Transmembrane	Reducted permeability	512 μg/mL	(Lu et al., 2016)
	Pro305Ala	Transmembrane	Reducted permeability	128 μg/mL	(Lu et al., 2016)
	Arg344Gly	NA	Reducted permeability	128 μg/mL	(Lu et al., 2016)
	Arg317His	NA	Reducted permeability	128 μg/mL	(Lu et al., 2016)
	Ala318Thr	NA	Reducted permeability	>128 μg/mL	(Lu et al., 2016)
	Pro327Thr	Transmembrane	Reducted permeability	128 μg/mL	(Lu et al., 2016)
	Leu338Trp	Transmembrane	Reducted permeability	>128 μg/mL	(Lu et al., 2016)
**UhpT**	Arg165Gly	Transmembrane	Reducted permeability	256 μg/mL	(Lu et al., 2016)
	Arg171Val	Transmembrane	Reducted permeability	>128 μg/mL	(Lu et al., 2016)
	Leu178Phe	Transmembrane	Reducted permeability	256 μg/mL	(Lu et al., 2016)
	Gly196Glu	Transmembrane	Reducted permeability	128 μg/mL	(Lu et al., 2016)
	Ala252Pro	NA	Reducted permeability	128 μg/mL	(Lu et al., 2016)
	Ser266Pro	Transmembrane	Reducted permeability	512 μg/mL	(Lu et al., 2016)
	Ile282Leu	NA	Reducted permeability	512 μg/mL	(Lu et al., 2016)
	Lys286Arg	NA	Reducted permeability	128 μg/mL	(Lu et al., 2016)
	Ala301Gly	Transmembrane	Reducted permeability	>128 μg/mL	(Lu et al., 2016)
	Arg312Pro	Transmembrane	Reducted permeability	128 μg/mL	(Lu et al., 2016)
	Glu317Lys	Transmembrane	Reducted permeability	128 μg/mL	(Lu et al., 2016)
	Gln320Lys	NA	Reducted permeability	512 μg/mL	(Lu et al., 2016)
	Arg323Lys	Transmembrane	Reducted permeability	512 μg/mL	(Lu et al., 2016)

NA, Not Available.

### Permeability impairment

#### GlpT system

Impairment in GlpT activity is one of the most common mechanisms of FOS^R^. Strains defective in GlpT transport are not able to grow using G3P as sole carbon source (Aghamali et al., 2019). In literature, there are several reports of common mutations in GlpT associated with reduced permeability and thus increased FOS MICs (**Table 1**). The deletion and/or truncation in GlpT protein are associated with reduction in permeability and loss of function in *E. coli* strains (Li et al., 2015; Ohkoshi et al., 2017). In 2020 Sorlozano-Puerto and colleagues investigated the effect of several mutations in GlpT from *E. coli* clinical isolates from Spain. The biological impact of such mutations was predicted through bioinformatic tool and tested by carbon grow test. The study identified possible alterations with a deleterious effect on GlpT activity, such as Gly84Asp, Pro212Leu, Leu373Arg, and thus a direct involvement in FOS^R^ (Sorlozano-Puerto et al., 2020) (**Table 1**). Differently, deletion W28del occurring in GlpT has been associate to FOS MICs >128 μg/mL in clinical ST131 *E. coli* from clinical setting in Czech Republic (Mattioni Marchetti et al., 2023). Another study evaluated mutations in GlpT from ESβL-producing *K. pneumoniae* from hospitals in Taiwan. In this study, Lu and colleagues identified several single amino acid substitutions, occurring in the transmembrane domains, such as Arg206Lys, Ile266Ser and Ile293Phe and associated with FOS resistance at high levels (FOS MICs = 256 μg/mL) (Lu et al., 2016) (**Table 2**).

#### UhpT system

Similar to GlpT, mutations in UhpT are likely to reduce G6P entry inside bacterial cell and thus FOS permeability. Indeed, the complete loss of UhpT peptide leads to the complete loss of the transport function and leads to FOS^R^ at high levels (FOS MICs >128 μg/mL) (Takahata et al, 2010; Li et al., 2015; Ohkoshi et al., 2017; Falagas et al., 2019). Different mutations have been reported in both *E. coli* and *K. pneumoniae* clinical strains, occurring in both transmembrane and topological domain, associated with a wide MICs range of FOS^R^ (64 μg/mL - 512 μg/mL) (Tseng et al., 2015; Seok et al., 2020; Ortiz-Padilla et al., 2022). Interestingly, Ballestero-Téllez and coauthors described the *in vitro* effect of premature Gln345stop in UhpT, which showed FOS MICs higher than 1,024 μg/mL in *E. coli* (Ballestero-Téllez et al., 2017).

#### UhpABC system

Impairment in the activity of UhpABC system might reduce the effectiveness of bacterial transportation systems and, consequently, reduce FOS influx into the bacterial cell (Kadner and Shattuck-Eidens, 1983). The loss of entire UhpA portion leads to different extent of FOS^R^ (MIC > 32 μg/mL) (Ohkoshi et al., 2017; Falagas et al., 2019), while deletion of 163-188 aa or premature stop codon in UhpA contribute to high level of FOS^R^ (MIC = 1,024 μg/mL) (Lucas et al., 2017; Ohkoshi et al., 2017). A study conducted by Cattoir et al. demonstrated the *in vitro* effect of mutations Gly469Arg in UhpB and Phe384Leu in UhpC. Both alterations showed a loss of function in the two regulators’ activity and an increased FOS MICs to resistance range (MIC = 128 μg/mL) (Cattoir et al., 2020). Another study conducted in 2017 highlighted the *in vitro* effect of mutations in UhpB (Thr27Stop, Gln262Stop, Trp181Stop, Leu255Stop, MIC = 1,024 μg/mL) and UhpC (Thr72Pro, = 1,024 μg/mL) in selected *E. coli* single-gene deletion mutants (Δ*glpT*, Δ*uhpT*, Δ*cyaA* and Δ*ptsI*) (Ballestero-Téllez et al., 2017)(**Table 1**).

#### Regulation in cAMP levels

Despite the relevant implication of CyaA activity in GlpT and UhpT expression, investigation of mutations in CyaA and its eventual effect on FOS MICs are still not clear, with just few reports conducted in *E. coli* strains (Yang et al, 2016; Ohkoshi et al., 2017) (**Table 1**).

## Acquisition of antibiotic resistance genes

### FosA family

**Figure 5:**
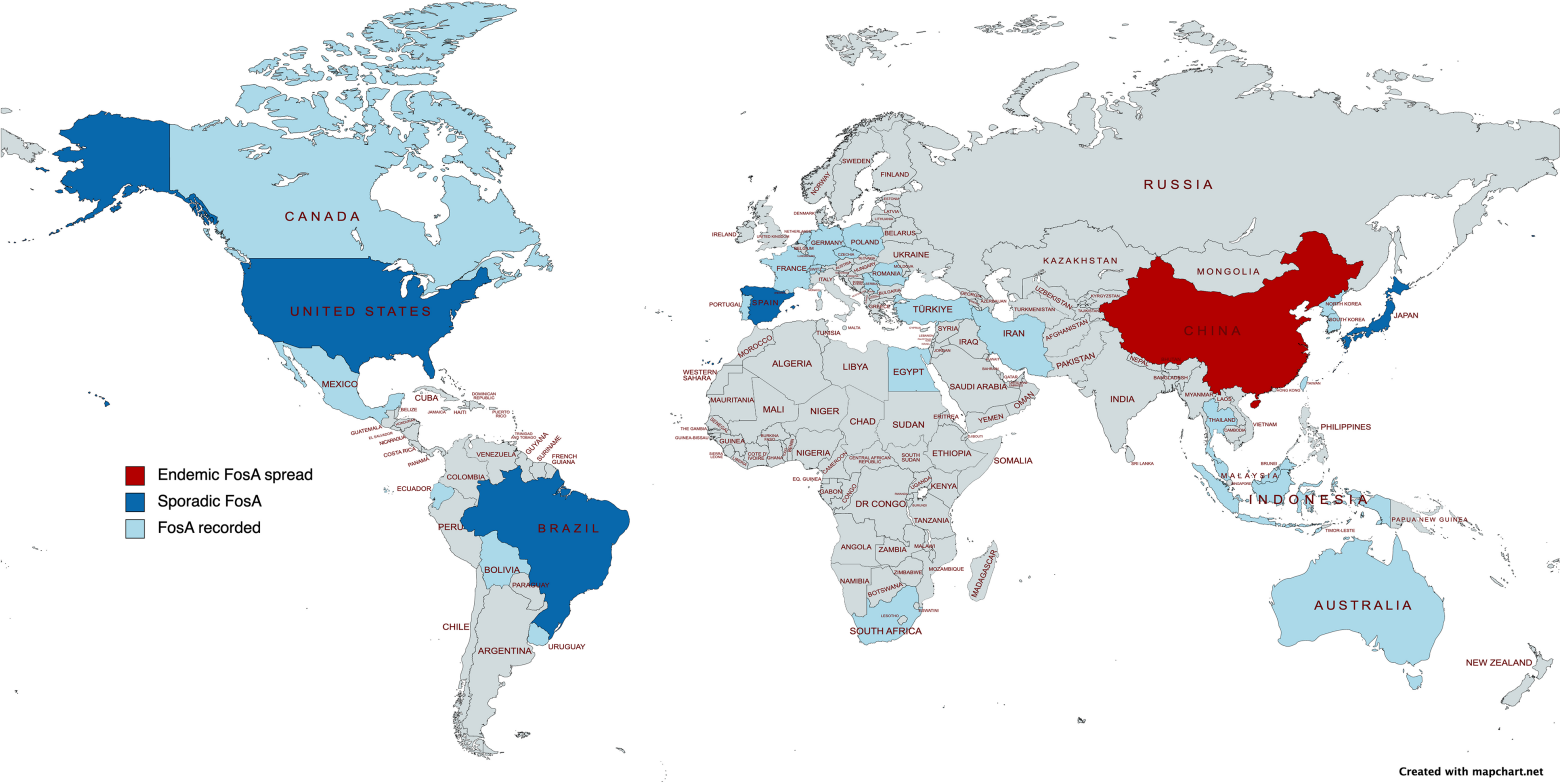
Epidemiological map of FosA among *Enterobacterales*. Created with mapchart.net.

**Figure 6:**
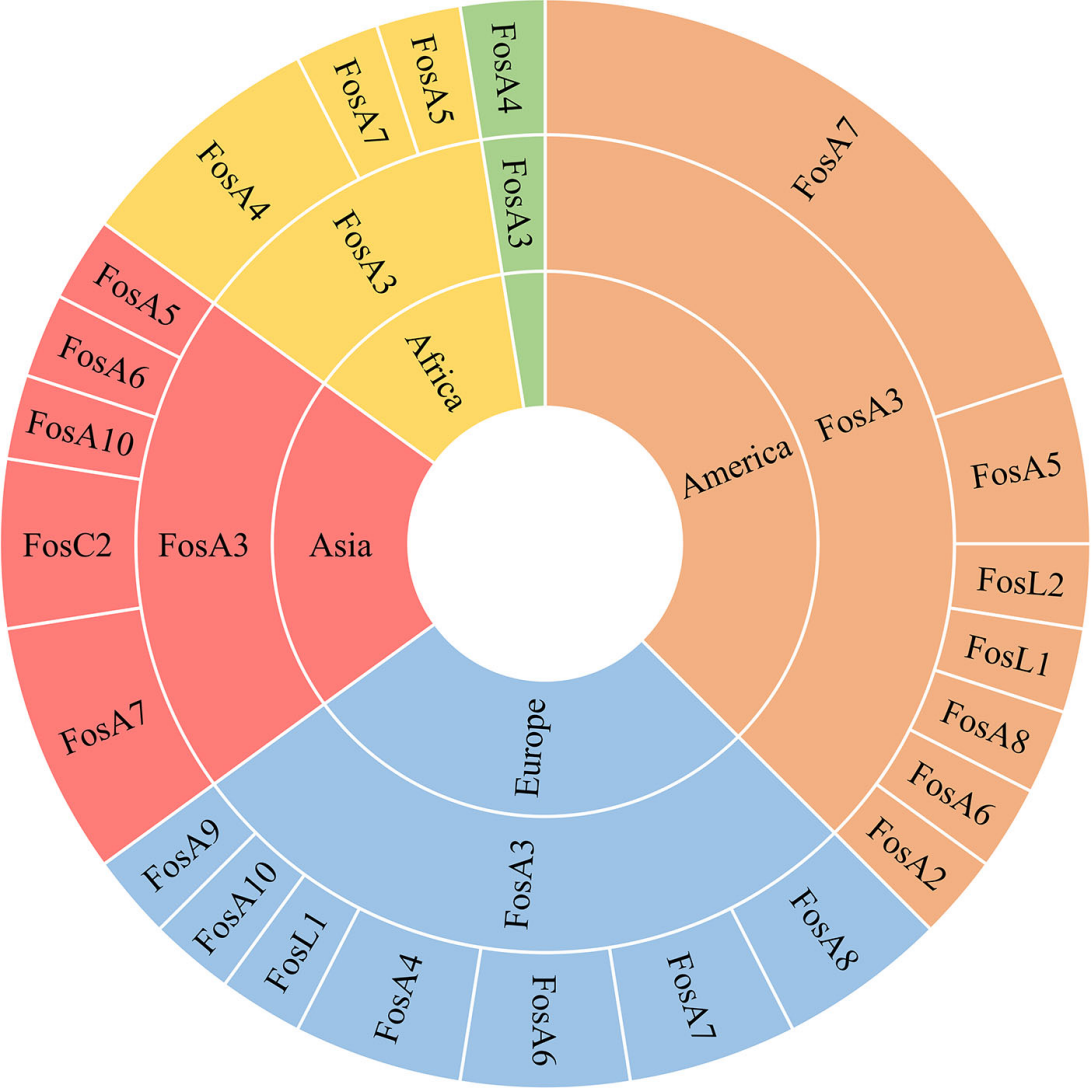
Hierarchy representation of FosA/C2/L1 prevalence in the continents Africa (yellow), America (orange), Asia (red), Europe (light blue) and Oceania (green).

**Figure 7:**
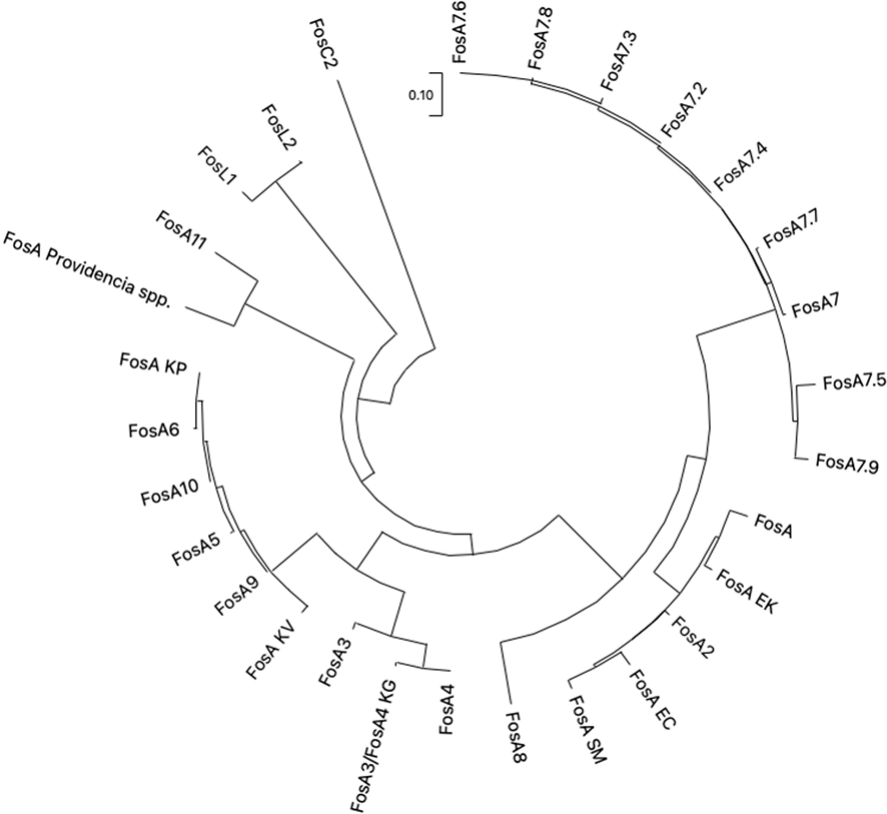
The evolutionary analysis and phylogenetic tree of FosA/C2/L1-2 proteins found in *Enterobacterales* were inferred by using the Maximum Likelihood method and ITT matrix-based model using MEGA 11. FosA KP (CDO16183.1), FosA KV (AWG41960.1), FosA EC (AWG41971.1), FosA EK (VAX69325.1), FosA P (WP_154635598.1), FosA SM (QOW96986.1), FosA2 (WP_025205684.1), FosA3 (WP_014839980.1), FosA4 (WP_034169466.1), FosA4 KG (WP_064548962.1), FosA5 (WP_012579083.1), FosA6 (WP_069174570.1), FosA7 (WP_000941934.1), FosA7.2 (WP_000941935.1), FosA7.3 (WP_023231494.1), FosA7.4 (WP_023216493.1), FosA7.5 (WP_000941933.1), FosA7.6 (WP_061377147), FosA7.7 (WP_058653118.1), FosA7.8 (WP_079820715.1), FosA7.9 (WP_071684814.1), FosA8 (WP_063277905.1), FosA9 (WP_114473955.1), FosA10 (WP_004214174.1), FosA11 (QZL11398.1), FosL1 (WP_161667239.1), FosL2 (WP_188331883.1). KP = *K. pneumoniae*, KV = *K. variicola*, KG = *K. georgiana*, EK = *E. kobei*, SM = *S. marcescens*, EC = *E. cloacae*.

FosA group is a class of metalloenzymes able to disrupt the epoxide ring of FOS drug. It depends on manganese (II) and potassium as cofactors, and on glutathione (GSH) as nucleophilic molecule. Nowadays, 11 different and genetically related variants have been deposited in GenBank Database and 10 of these are reported in the global dissemination *scenario* (**Figures 5**–**7**). In accordance with Ito et al., 2017, *fosA* genes are chromosomally distributed in *Providencia stuartii*, *Providencia rettgeri*, *K. pneumoniae*, *Klebsiella oxytoca*, *Serratia marcescens*, *Enterobacter aerogenes* and *Enterobacter cloacae* genomes, while they are rarely reported in *E. coli*, *Citrobacter freundii, Proteus mirabilis* and *Acinetobacter baumannii* (Zurfluh et al., 2020).

## FosA and FosA2

The first plasmid-mediated *fosA* was identified and isolated from a clinical sample of *S. marcescens* in Spain in 1980 (Mendoza et al., 1980) (**Figure 1**). *FosA* was located on a Tn*2921* cassette on the plasmid pSU912 (Seoane et al., 2010) (**Figure 7A**). The origin of FosA is linked with the FOS-modifying enzyme Fos^EC^, located on *E. cloacae* chromosome (100% identity) (García-Lobo and Ortiz, 1982; Ito et al., 2017).

FosA2 variant was first reported in 2011 (Xu et al., 2011) in *E. cloacae* chromosome from a water sample in Canada (**Figure 1**). Currently, *fosA2* reports are correlated with chromosomal location only.

## FosA3

**Figure 8:**
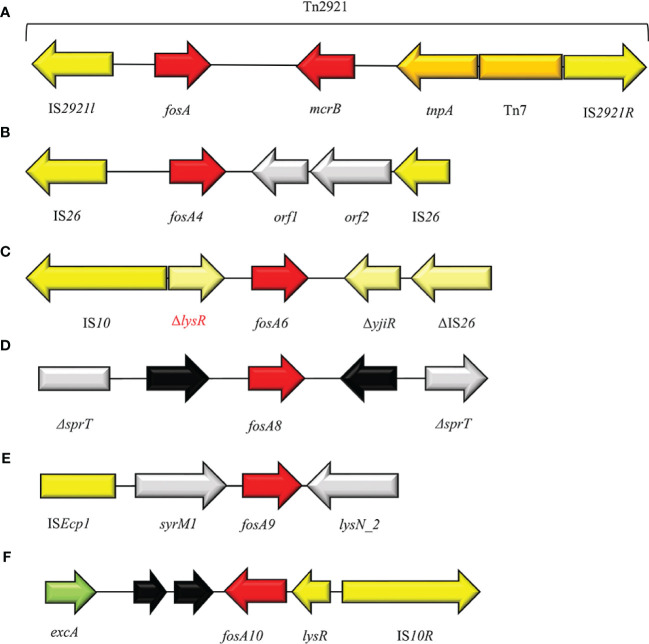
Structure of representative genetic environments of **(A)** *fosA* (FJ829469.1), **(B)** *fosA4* (Loras et al., 2021), **(C)** *fosA6* (KU254579.1), **(D)** *fosA8* (SAMN12496803), **(E)** *fosA9* (Wang et al., 2019), **(F)** *fosA10* (MT074415.1). Yellow = IS, light yellow = deleted IS, red = antimicrobial resistance genes, gray = open-reading frame, black = unknown proteins, green = surface exclusion protein.

**Figure 9:**
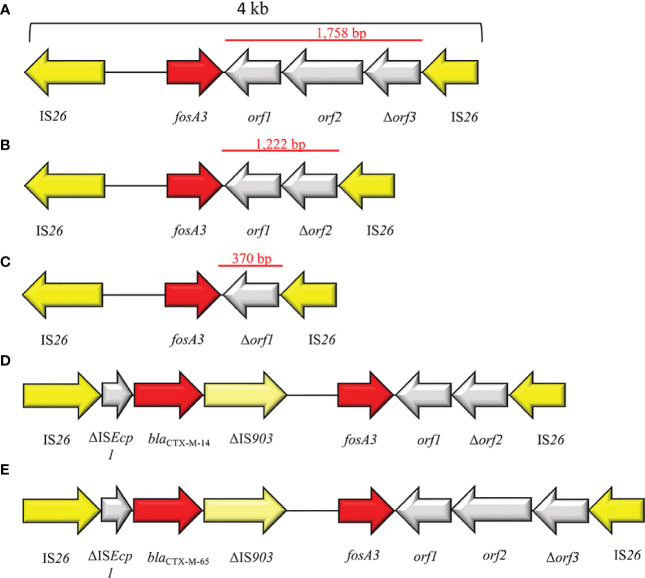
Representation of genetic environments of *fosA3*. **(A)** (JQ432559), **(B)** (JX442752), **(C)** (JX442751), **(D)** (JQ823170), **(E)** (JX442753). Yellow = IS, light yellow = deleted IS, red = antimicrobial resistance genes, gray = open-reading frame.

FosA3 is the plasmid-acquired subtype mostly disseminated and reported (**Figure 8**). FosA3 shows close relation (>94% identity) to the chromosomally encoded FosA^KG^ from *Kluyvera georgiana*. The first report is dated 2010 from a clinical isolates *E. coli* in Japan (**Figure 1**). Shortly after, in 2013, a *fosA3* plasmid-mediated dissemination among food-chain animals in Chinese region was reported (Hou et al., 2012; Ho et al., 2013). Currently, China has the highest dissemination of plasmid-mediated *fosA3* among both clinical and veterinary settings (**Figures 5**, **8**). Concerning Chinese veterinary field, several animal species have been identified as silent reservoir, ranging from pets, as dogs and cats, to food-chain animals, as pigs and bovines, and wild animals, as pigeons. *FosA3* is organized in a composite transposon, of 4 kb in size, consisting in two IS*26* elements with the same orientation, that flank the cassette *fosA3-orf1-orf2-Δorf3* (Wachino et al., 2010) (**Figure 9A**). *fosA3* genes are located 316 bp downstream of IS*26*, while the spacer region between the 3′ end of *fosA3* and IS*26* can varies in size (1,758 bp, 536 bp and 370 bp) (**Figure 9A**). Interestingly, the 1,758 bp region shows 79% nucleotide identity with part of *K. pneumoniae* strain 342 chromosome (Hou et al., 2012). Based on the transposon composition, five major *fosA3* environment can be classified in *Enterobacterales* (named type A-E): A) IS*26-fosA3-orf1-orf2-*Δ*orf3-*IS*26*, B) IS*26-fosA3-orf1-*Δ*orf2-*IS*26*, C) IS*26-fosA3-*Δ*orf1-*IS*26*, D) IS*26*-IS*Ecp1*-*bla*_CTX-M-14_-ΔIS*903D-fosA3-orf1-*Δ*orf2-*IS*26*, E) IS*26*-IS*Ecp1*-*bla*_CTX-M-65_-ΔIS*903D-fosA3-orf1-orf2-*Δ*orf3-*IS*26* (**Figures 9A–E**). The type A is the predominant type, and it is associated to IncF, IncI1, IncN, IncB/O and several untypable plasmids (Ho et al., 2013; Liu et al., 2022). *FosA3* can be easily co-harbors on the same plasmid with other ESβLs, as *bla*_CTX-M-3_, *bla*_CTX-M-8_, _-14_, _-55_, _-65_ and _-123_ (Ho et al., 2013; Mazé et al., 2014; Xie et al., 2016; Yang et al, 2016; Dantas Palmeira et al., 2018). The first dissemination of FOS^R^ in several *E. coli* strains from veterinary settings was reported by Hou et al. (2012). The study identified *E. coli* isolates co-harboring *fosA3* and *bla*_CTX-M-65_ on IncF plasmids. The *fosA3* cassette consisted of *fosA3* transposon Type B (**Figure 9B**), with a spacer region between the 3’ end of *fosA3* and IS*26* of 536 bp (Hou et al., 2012).

In Korea, a point prevalence study highlighted the presence of seven *Enterobacteriaceae* strains co-producing FosA3 and CTX-M out of 347 ESβL producers. All the seven strains harbored *fosA3* + *bla*_CTX-M-like_ in the same IS*26*-composite transposon (Sun et al., 2012). Ho et al. in 2013 evaluated the dissemination of plasmid-mediated *fosA3* gene among animals and humans, highlighted 97 FosA3-producing *E. coli* strains out of 1,693 (Ho et al., 2013). Wei Jiang et al. screened 234 CTX-M-producing *E. coli* isolates collected from chickens from 2014 to 2016 in China and identified 64 *fosA3*+*bla*_CTX-M-like_ positive *E. coli* located on IncFII, IncI1, IncHI2 and IncB/O. Additionally, the authors identified two novel genetic environments surrounding the *fosA3* (IS*Ecp1*-*bla*_CTX-M-65_-ΔIS*903D*-IS*26*-*fosA3*-*orf1*-*orf2*-*Δorf3*-IS*26* and IS*26*-IS*Ecp1*-*bla*_CTX-M-3_-*orf477*-*bla*_TEM-1_-IS*26*-*fosA3*-*orf1*-*orf2*-Δ*orf3*-IS*26*) (Jiang et al., 2017). In *E. coli*, genomic studies highlighted the occurrence of FosA3 in ST10 (Seok et al., 2020), ST12 (Hameed et al., 2022), ST38 (Norizuki et al., 2018; Hameed et al., 2022), ST 46 (Hameed et al., 2022), ST57 (Hameed et al., 2022), ST69 (Hameed et al., 2022; Liu et al., 2022), ST95 (Hameed et al., 2022), ST106 (Seok et al., 2020), ST117 (Fernandes et al., 2018; Zhao et al., 2022), ST131 (Galindo-Méndez et al., 2022; Hameed et al., 2022), ST1193 (Hameed et al., 2022), ST1196 (Hameed et al., 2022), ST2736 (Wang et al., 2018), ST7584 (Hameed et al., 2022), ST10184 (Hameed et al., 2022), ST11350 (Ewbank et al., 2022), ST648 (Yang et al, 2016), ST156 (Xie et al., 2016). The occurrence of plasmid-mediated *fosA*-like genes turns out to be worrying in ST131 clone due to its virulence and pathogenic features (Forde et al., 2019; Galindo-Méndez et al., 2022; Hameed et al., 2022). Indeed, ST131 is a globally dominant MDR clone associated with UTI, and it is involved in the global dissemination of ESβLs as CTX-M-15 type (Forde et al., 2019; Jafari et al., 2020). However, from 2014 to 2018 Liu and colleagues evaluated the prevalence of mobile *fosA3* gene in ducks from 23 Chinese farms and they highlighted the predominance of the ST69 as *fosA3*-harboring clones among *E. coli* strains (Liu et al., 2022). Similarly, Loras et al. identified an ST69 *E. coli* strain from urine sample in Spain and co-harboring *fosA3*+*bla*CTX-M-55 (Loras et al., 2021). Another clinical case of *E. coli* ST69 harboring a plasmid-mediated *fosA3* (IncN) was identified in Uruguay from pediatric UTI cases (Garcia-Fulgueiras et al., 2022). ST69 is an emerging Extraintestinal Pathogenic *E. coli* (ExPEC) lineage detected in both humans and animal settings, that is globally involved in UTI from both the community and the hospital environment (Giacobbe et al., 2015; Hammad et al., 2019). *E. coli* ST69 was originally susceptible to almost all the antibiotic families, but the acquisition of β-lactams and FOS^R^ traits could affect the use of FOS in UTI treatment (Doumith et al., 2015; Garcia-Fulgueiras et al., 2022).

First isolation of plasmid-mediated *fosA3* in clinical *K. pneumoniae* strains was in 2012, when Lee and co-authors described the co-presence of *fosA3*+*bla*_CTX-M-14_ on an IncN plasmid and organized in IS*26*-IS*Ecp1*-*bla*_CTX-M-14_-ΔIS*903D*-IS*26*-*fosA3*-*orf1*-*orf2*-*Δorf3*-IS*26* (with a spacer sequence of 1,222 bp) (Lee et al., 2012). Lately, in 2015, Jiang Y et al. reported the characterization of 94 KPC+FosA3 co-producing *K. pneumoniae* collected from twelve Chinese hospitals. Additionally, the authors highlighted a clonal relation between KPC- and FosA3-producers, indicating a FOS^R^ clonal dissemination in China (Jiang et al., 2017). In *K. pneumoniae* plasmid-mediated *fosA3* is largely associated with isolates belonging to ST 11 (Xiang et al., 2015; Nishida et al., 2020), ST37 (Taniguchi et al., 2017), ST485 (Zhou et al., 2022). In recent years, a secondary spread of plasmid-mediated *fosA3* occurred in *Salmonella* spp. among food-chains animals and humans in China (Wang et al., 2020; Zhang et al., 2020). Outside Chinese settings, similar cases have been recorded from pediatric patients in Spain (Vázquez et al., 2022), from clinical patients in USA (Turcotte et al., 2022), and from a wild bird in Germany (Villa et al., 2015). Noteworthy, Villa and colleagues described the first case of a *Salmonella enterica* Serovar Corvallis co-producing FosA3+NDM-1+CMY-16. *FosA3* and *bla*_NDM-1_ were located on the same IncA/C2 plasmid and *fosA3* included in a type A transposon (Villa et al., 2015). This report highlighted the bird’s migration as route for environmental diffusion of *fosA3* from norther Asia to Europe (Villa et al., 2015). Among *Salmonella* spp. strains, transposon Type A is the most spread *fosA3* environment, located on IncFII (Lin and Chen, 2015) and IncHI2 (Wong et al., 2016), followed by Type C on IncFIB (Vázquez et al., 2022) and type D on IncHI2 (Wong et al., 2016). Interestingly, a multi-replicon IncC-IncN plasmid, coharboring *fosA3* Type A and *bla*_CTX-M-14_ have been already isolated from chickens in China (Zhang et al., 2020). FosA3 cases occurred in *Salmonella* ST32 (Vázquez et al., 2022), ST17 (Wang et al., 2022), ST34 (Wang et al., 2022), ST198 (Wang et al., 2022). Since 2017, few reports evaluate the occurrence of plasmid-mediated *fosA3* in *P. mirabilis* from both hospitalized patient and food-chain animals (He et al., 2017; Hua et al., 2020; Lei et al., 2020). The first case focused on the chromosomal integration of *bla*_CTX-M-14_/*bla*_CTX-M-65_ and *fosA3* in *P. mirabilis* collected in 2015 from diseased broilers in China, with the following compositions: a) IS*26*–ΔIS*Ecp1*–*bla*_CTX-M-14_–ΔIS*903*–*fosA3*–1,222 bp–IS*26;* b) IS*26*–Δ*traI*–*fip*–ΔIS*Ecp1–bla*_CTX-M-65_–IS*903D*–*iroN*–IS*26*–*fosA3*–1758 bp–IS*26.* In the same study, the presence of the transposition unit “b” was detected in IncHI2 plasmid from *E. coli* ST117, together with the presence of minicircles that contain *fosA3*, *bla*_CTX-M-65_ and IS*26* (He et al., 2017). Thus, the authors speculated the *fosA3+bla*_CTX-M-65_ integration into the *P. mirabilis* chromosome *via* a transposable minicircle from *E. coli* (He et al., 2017). Similarly, the presence of minicircles harboring IS*26* and *fosA3* was identified even in *S. enterica* from a Chinese chicken and speculations about their role in *fosA3* acquisition and spread are under evaluation (Zhang et al., 2020). Similar environments containing *bla*_CTX-M-65_ + *fosA3* were identified in retail meat and aquatic products from markets (Ma et al., 2022), from diseased pig (Lei et al., 2018; Song et al., 2022) and from retail chickens (Lu et al., 2021) from Chinese regions, while the co-expression CTX-M-3+FosA3 was reported from Chinese chicken (Turcotte et al., 2022). Rather worrying was the isolation of a KPC-2+CTX-M-65+FosA3 producing *P. mirabilis* from a Chinese 49-year-old female with a pulmonary infection (Hua et al., 2020). The *bla*_CTX-M-65_+*fosA3* was located on an IncFII-33 and the authors emphasized the successful association of IS*26* and IncFII-33 in spreading antimicrobial resistance features (Hua et al., 2020).

*FosA3* easily fits in different plasmid environments, including single- and multi-replicons. The major vehicle of plasmid-mediated *fosA3* spread is IncFII (Hou et al., 2012), followed by IncI1 (Sato et al., 2013), IncN (Liu et al., 2022), IncHI2 (Chen et al., 2021), and IncP (Hameed et al., 2022). The successful and global diffusion of *fosA3* could be explain by the combination of IS*26* sequences and IncFII plasmids. *FosA3* genes are mainly flanked by IS*26*, that play a fundamental role in AMR effective transposition and in their AMR dissemination among *Enterobacterales* (Partridge et al., 2018; Lv et al., 2020). Moreover, as mentioned elsewhere, IS*26*-flanked transposons are able to form circular intermediates that could accelerate the spread of *fosA3* (He et al., 2015; Harmer and Hall, 2016). The IncFII plasmids are commonly low copy number plasmids and are recognized as vehicles of ESβLs dissemination among *Enterobacterales* (Muthuirulandi Sethuvel et al., 2019). Moreover, researchers speculate on the role of IncFII F33:A-:B- and F2:A-:B- in *fosA3* dissemination due to its high adaptation levels (Hou et al., 2012; Sun et al., 2012).

European epidemiology of *fosA3* is still low, with few reports from clinical *E. coli* strains in Spain (Loras et al., 2021), from clinical settings in Switzerland (Mueller et al., 2019), from veterinary and enviroment in Germany (Freitag et al., 2018), from clinical and veterinary settings in France (Benzerara et al., 2017; Lupo et al., 2018) and in Portugal (Mendes et al., 2016). Although reports in literature highlight a predominant association of *fosA3* with *bla*_CTX-M-like_ genes, recent studies revealed an emerging co-presence with carbapenemases in *E. coli* (Zhao et al., 2015; Peng et al., 2019), *K. pneumoniae* (Xiang et al., 2015; Singkham-In et al., 2020; Hao et al., 2021), *C. freundii* (Feng et al., 2015), *E. cloacae* (Hameed et al., 2022), *Cronobacter sakazakii* (Liu et al., 2017) and *S. enterica* (Villa et al., 2015). In literature, reports highlighted the co-expression of FosA3 and carbapenemases such as FosA3+KPC (Shi et al., 2018), FosA3+NDM (Tian et al., 2020), FosA3+VIM (Tang et al., 2020), FosA3+OXA-48 (Singkham-In et al., 2020), FosA3+KPC+IMP (Tseng et al., 2017; Peng et al., 2019), FosA3+KPC+NDM (Peng et al., 2019), FosA3+NDM+OXA-48 (Singkham-In et al., 2020).

In the last six years, the co-presence of EsβL+*fosA3*+*mcr*-like genes has been already detected in both clinical and veterinary environment (Birgy et al., 2018; Hoang et al., 2022). This combination of multi-resistance strains was reported in China (Zhao et al., 2018), France (Birgy et al., 2018) and Ecuador (Hoang et al., 2022). Worryingly, the co-expression of FosA3+MCR-1 and NDM-1/KPC-2 among *E. coli* strains has been already identified in hospitalized patients and food-chain animals (Zhao et al., 2018; Peng et al., 2019; Tian et al., 2020). Liu and colleagues described the occurrence of MCR-1+FosA3+NDM-like in several *E. coli* strains collected from chicken farm in China (Liu et al., 2017). The study identified the presence of *i*) MCR-1+NDM-9+FosA3 coproducing ST10; *ii*) MCR-1+NDM-4+FosA3 co-producing ST117; *iii*) MCR-1+NDM-1/-9+FosA3 co-producing ST156; *iv*) MCR-1+NDM-4/-9+FosA3 co-producing ST297; *v*) MCR-1+NDM-9+FosA3 co-producing ST2973 (Liu et al., 2017). During a surveillance study in 2015 among animal farms in Shandong, two pan-drug strains of *C. sakazakii* were isolated from sick chickens (Liu et al., 2017). The study clarified the copresence of *bla*_NDM-9_+*fosA3*, located on the same conjugative IncB/O plasmid, and *mcr-1* on a IncI2 plasmid (Liu et al., 2017).

## FosA4

FosA4 enzyme shows 94% amino acid identity with FosA3, and speculation proposes *Kluvyera georgiana* as possible origin of the plasmid-mediated resistance gene *fosA4* (Nakamura et al., 2014; Rodriguez et al., 2018). FosA4 epidemiology is limited and varies geographically, but it was mainly reported in *E. coli* isolates (**Figure 8**). Increasing cases of FosA4-producing *E. coli* have been reported among food-chain animal settings in Egypt (Soliman et al., 2021; Sadek et al., 2022) and in France (Lupo et al., 2018). Other cases, concerning clinical settings, have been described from hospitals in Madrid (Loras et al., 2021) and Australia (Mowlaboccus et al., 2020). In Southern Turkey, Cansu Önlen Güneri and co-authors described a regional diffusion of plasmid-mediated *fosA4* among *E. coli* collected from waste-water treatment plant (Güneri et al., 2022). The *fosA4* gene has been reported predominantly on IncFII plasmid type and, consequently, on IncHI2 and IncI1 (Ma et al., 2015; Loras et al., 2021; Ramadan et al., 2021). IncFII and IncI1 normally harbors additional genes responsible for resistance to other antibiotics such as penicillins, sulphonamides and aminoglocosyde (Mowlaboccus et al., 2020; Ramadan et al., 2021). *FosA*4-harboring plasmids often coexist with *bla*_CTX-M_- and *mcr*-1-harboring plasmids (Ramadan et al., 2021; Sadek et al., 2022). FosA4 is associated with a conserved cassette of 4,022 bp in size, consisting of: two IS*26*, *fosA4*, tetR/acrR family and a helix-turn-helix domain. In southern Turkey, a novel genetic enviroment was detected, replacing the upper IS*26* with an IS*4* (Güneri et al., 2022) (**Figure 7B**). MIC data for *fosA4* have been reported in *E. coli* as >1,024 µg/ml (Güneri et al., 2022).

## FosA5

**Figure 10:**
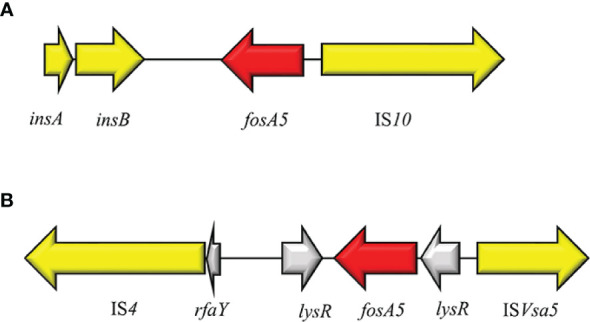
Structure of representative genetic environments of *fosA5*. **(A)** (KP143090), **(B)** (MH399264). Yellow = IS, light yellow = deleted IS, red = antimicrobial resistance genes, gray = open-reading frame.

In 2015, Ma Y et al. reported the first case of *fosA5* from a clinical *E. coli* strain in an inpatient with hospital-acquired pneumonia in China (Ma et al., 2015). FosA5 enzyme shares 69% amino acid sequence similarity with FosA and 80% with FosA3. The *K. pneumoniae* chromosome has been identified as the origin of *fosA5* variant and its spread is associated with pKP96 plasmid, as reported by Ho PL et al., 2013 (Xu et al., 2011). The genomic enviroment of *fosA5* is characterized by *insA* and *insB* and an IS*10* in the opposite side (**Figures 10A, B**). In 2019 Wang S and colleagues investigated the genomic enviroment of an IncHI2A plasmid (pIMP26) coharboring *bla*_IMP-26_, *bla*_DHA-1_ and *fosA5*, isolated from an *E. cloacae* strain involved in blood infection (Wang et al., 2019). In pIMP26, the *fosA5* structure was as follow: IS*4*, *rfaY*, *lysR*, *fosA5*, *rfaY*, IS*Vsa5* (IS*4*-like) (Wang et al., 2019). A similar organization of the *fosA5* cluster has been detected in an IncHI2/2A plasmid (pEHZJ1) from an *E. hormaechei* of clinical origin (Gou et al., 2020). FosA5-carrying *E. coli* strains were found to be highly FOS^R^ (MIC = 512 µg/ml) (Ma et al., 2015).

## FosA6

FosA6 was firstly described in a clinical CTX-M-2-producing *E. coli* ST410 from an US hospital in 2017 (Guo et al., 2016). *FosA6* was carried on a self-conjugative IncFII plasmid (69 kb) and inserted in the cassette IS*10R-*Δ*lysR*-*fosA6*-Δ*yjiR_1*- ΔIS*26*, nearly identical to those on the chromosomes of some *K. pneumoniae* strains (**Figure 7C**). Moreover, *fosA6* shared >99% sequence identity with chromosomally encoded *fosA* in *K. pneumoniae.* A point prevalence study conducted among seven Hospitals in Madrid, identified the only European case of ST354 *E. coli* producing FosA6 enzyme (Loras et al., 2021). *FosA6*-carrying *E. coli* had FOS MIC values of 128 to >1024 µg/ml (Guo et al., 2016).

## FosA7

In 2015 Dhanani and colleagues investigated the resistome of four FOS^R^ *S. enterica* serovars Heidelberg from broiler chickens among different commercial farms in Canada (Dhanani et al., 2015). As described later by Rehman et al., the 4 *S. enterica* strains produced a FosA-like enzyme, named FosA7, with a chromosomal location (Rehman et al., 2017).

**Figure 11:**
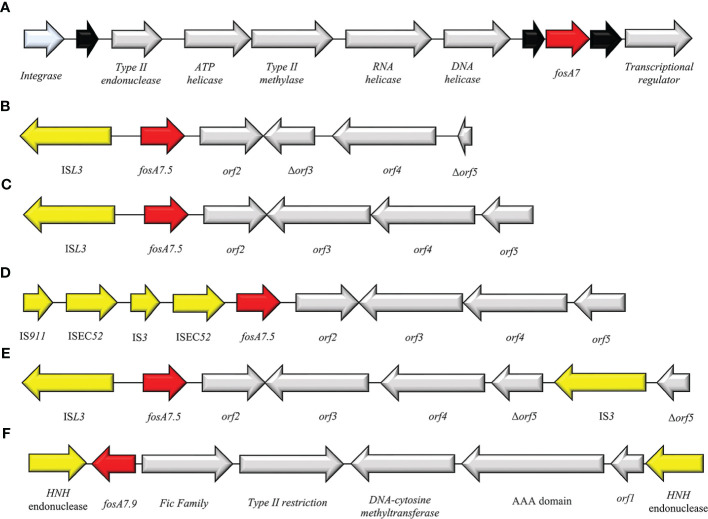
Structure of representative genetic environments of **(A)** *fosA7* (GCA_000973785.1) and *fosA7.5* **(B)** (OM355479), **(C)** (CP085638), **(D)** (CP085637), **(E)** (CP05525.1), **(F)** (CP047307). Yellow = IS, light blue = integrase, red = antimicrobial resistance genes, gray = open-reading frame, black = unknown protein.

Currently, 9 alleles of *fosA7* genes are deposited in GenBank (*fosA7.1*-*fosA7.9*). All these variants have a chromosome location among different bacterial species. *FosA7.5* and *fosA7.9* are strictly linked with the chromosome of *E. coli* and *C. freundii*, respectively. In *Salmonella* spp. *fosA7* is surrounded by two hypothetical proteins and located in an integrase cassette composed of Int-*type II endonuclease*-*ATP helicase*-*type II methylase*-*RNA helicase*-*DNA helicase* (**Figure 11A**). In *E. coli* the intercellular diffusion of *fosA7.5* is due to the composite transposon flanked by IS*L3* and IS*3* (IS*911* and IS*EC52*) elements (**Figure 11B–E**). A different composition has been highlighted for *fosA7.9* in *C. freundii*: the *fosA7.9* cassette is flanked by HNH endonuclease at both sides and organized in *HNH endonuclease*-*fosA7.9*-*Fic family*-*type II restriction*-*DNA methyltransferase*-*AAA domain*-*HNH endonuclease* (**Figure 11F**) (Mattioni Marchetti et al., 2023).

The epidemiology of FosA7 family displays a relevant dissemination, with reports in livestock animals, clinical settings and enviroment (Balbin et al., 2020; Jovčić et al., 2020; Mosime et al., 2022). The Canadian and USA regions reported the larger diffusion of *fosA7*, followed by China (Pan et al., 2021). Recently, cases of FosA7 enzymes have been described in South Africa from *Citrobacter koseri* (Ekwanzala et al., 2020), in Czech Republic from *C. freundii* (Mattioni Marchetti et al., 2023) and in Poland from *E. coli* (Skarżyńska et al., 2021). Expression of FosA7 showed high value of FOS^R^ MIC (>512 mg/ml) (Rehman et al., 2017).

## FosA8

The newly plasmid-encoded *fosA8* has been detected in clinical *E. coli* strains from a Swiss collection obtained from 2012 and 2013. The *fosA8* gene was located on a 50 kb IncN plasmid and flanked by two copies of deleted *sprT* gene. FosA8 shows the highest identity with the chromosomally encoded *fosA* of *Lecleria adecarboxylata* (Poirel et al., 2019) and 96% identity with FosA7.5 from *E. coli* (Milner et al., 2020). Recently, Biggel et al. described a FosA8-producing *K. pneumoniae*, isolated from food in Switzerland, on a 65.5 kb IncN-IncR plasmid and located in the cassette IS*26*-ΔIS*15*-*ardA–ccgC–ccgD–ccgEIII–ardR–ardB–mucA–mucB–*Δ*sprT–fosA8–orf1–*Δ*sprT–ardK–repA–orf2–*IS*26* (Biggel et al., 2021) (**Figure 7D**). FosA8 confers high resistance levels to FOS, with MIC > 1,024 µg/ml (Poirel et al., 2019).

## FosA9

FosA9 has been reported in 2019 by Doesschate et al. from an *E. coli* strain causing bacteremia in Utrecht. The patient had suffered from recurrent episodes of sepsis, with blood cultures positive for *K. variicola*, which was identified as the source of *fosA9*. The *fosA9* genomic environment consisted of a IS*Ecp1*-*syrM1*-*fosA9*-*lysN2* region, flanked by 5 bp DRs (AAAAA) and identical to those found in *K. variicola* (Wang et al., 2019) (**Figure 7E**). The expression of FosA9 confers FOS^R^ at high levels, with MIC > 1,024 µg/ml (Ten Doesschate et al., 2019).

## FosA10

The FosA10 enzyme has been described by Ying Huang et al. from a local broiler meat outlet in Pakistan. A 53,736 bp IncFII plasmid harbored the *fosA10*, inserted in a 4,328 bp variable region, flanked by two copies of IS*10* element (Huang et al., 2020) (**Figure 7F**). Differently, the identical genomic enviroment was identified on a IncK plasmid from a clinical ST648 NDM+FosA10-producing *E. coli* isolated in Czech Republic (Mattioni Marchetti et al., 2023). FosA10 shares highest identity with FosA6 and FosA9 (ID = 97.84%), confirming its possible origin from *K. pneumoniae* species (Huang et al., 2020). In *E. coli* strains FosA10 induces FOS^R^ phenotype with MIC >128 µg/ml (Huang et al., 2020).

## FosC2

**Figure 12:**
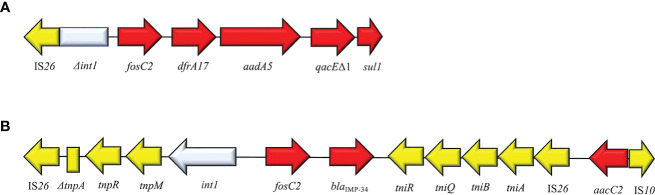
Structure of representative genetic environments of *fosC2*. **(A)** (AB522969) (Lucas et al., 2017), **(B)** (KM877517) (Guo et al., 2016). Yellow = IS, light blue = integrase, red = antimicrobial resistance genes, gray = open-reading frame, black = unknown protein.

FosC2 is a metalloenzyme able to induce resistance profiles to FOS and shared a 56% sequence identity with FosEC of *E. cloacae*. It was identified for the first time in 2010 from clinical *E. coli* in Japan. FosC2 disseminates *via* plasmid but is rarely reported. Originally, *fosC2* was described in integron type I structure: IS*26*-Δ*IntI1*-*fosC2*-*dfrA17*-*aad5*-*qacEΔ1*-*sul1* (Wachino et al., 2010) (**Figure 12**). Subsequently, in 2015 Wang and colleagues reported the second clinical case of *fosC2* disseminated *via* plasmid in a carbapenemases-producing *E. cloacae* strain. The plasmid (pIMP-HB623) was classified as IncL/M1 and harbored the composite cassette IS*26*-Δ*tnpA*-*tnpR*-*tnpM*-*IntI1*-*fosC2*-*bla*IMP34-*tniR*-*tniQ*-Δ*tniA*-IS*26* (Wang et al., 2015). Both cases reported in literature, emphasized the co-expression of FosC2 and ESβLs. Recently, speculation on FosC2 recognizes *Aliidiomarina shirensis* as a possible progenitor for plasmid-mediated *fosC2* (Ortiz de la Rosa et al., 2022). FosC2 expression induces broad FOS^R^ profile (MIC value = 128 µg/ml).

## FosL1 and FosL2

**Figure 13:**
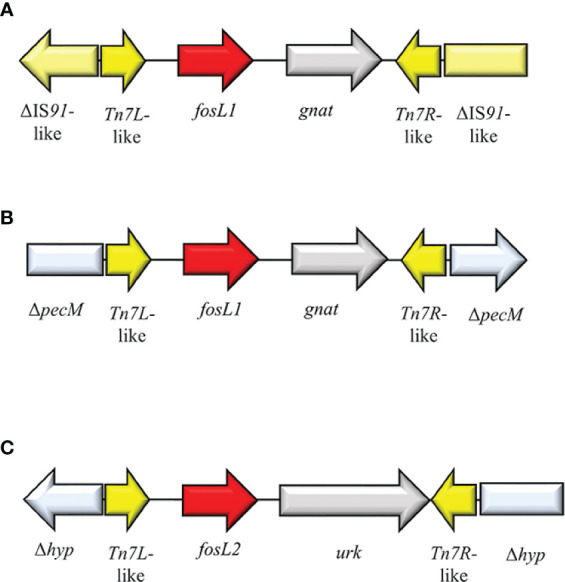
Structure of representative genetic environments of **(A, B)** *fosL1* (MN464149, SAMN11620633) (Rehman et al., 2017) and **(C)** *fosL2* (SAMN11027629) (Rehman et al., 2017). Yellow = IS, light blue = integrase, red = antimicrobial resistance genes, gray = open-reading frame.

FosL1 is a novel glutathione *S*-transferase metalloenzyme that shared a 63% identity with FosA8. FosL1 was described on a conjugative IncX1 plasmid in a *E. coli* strain of a Swiss patient (Kieffer et al., 2020). The genomic enviroment surrounding *fosL1* consisted of a mobile insertion cassette, flanked by ΔIS*91*-like at both sides. The same *fosL1* cassette, was detected on an IncQ1 plasmid from a clinical *S. enterica*. Subsequently, an *in-silico* analysis of *fosL1* identified a similar gene, classified as *fosL2*, on an IncP-like plasmid, collected from a clinical *S. enterica* strain. Genomic environment of *fosL2* consisted of Tn*7L*-like-*fosL1*-*urk*-Tn*7R*-like and flanked by Δ*hyp* at both sides (Kieffer et al., 2020) (**Figure 13**). The ancestor source for FosL1-2 remains unknown. FosL1 induces FOS^R^ profile at high level (MIC = 1,024 µg/ml) (Kieffer et al., 2020).

## Epidemiological breakpoints and detection strategies

According to European Committee on Antimicrobial Susceptibility Testing (EUCAST) and the Clinical and Laboratory Standards Institute (CLSI), agar dilution method (ADM) is the gold standard for FOS MIC detection in both Gram-positive and -negative bacteria but the breakpoints for FOS susceptibility have been formalized for few species and are different for CLSI and EUCAST. EUCAST breakpoints for *Enterobacterales* define as susceptible (S) MIC ≤ 32 mg/L and resistant (R) MIC > 32 mg/L, while CLSI breakpoints for *E. coli* are S ≤ 64 mg/L, I =128 mg/L, R ≥ 256 mg/L (Falagas et al., 2008).. Currently, there is a lack of fast, time-saving susceptibility tests for FOS and the limited breakpoints standardization, that highlights the difficulty in monitoring FOS profiles epidemiology and in identifying FOS^R^ strains. In this section we describe the current available methods for the investigation of FOS susceptible profiles among *Enterobacterales*.

### Agar dilution method (ADM)

The reference method ADM consists in the incorporation of different concentration of FOS (generally from 0.25 mg/ml up to 1,024 mg/ml) into Mueller-Hilton (MH) agar, added with 25 mg/L of G6P; Balouiri et al., 2016). Then, a 0.5 MacFarland suspension of the studied strain is prepared and diluted, to obtain the final inoculum required of 1 × 104 CFU/spot (2 μl). When replicators with 1-mm pins that deliver 0.1 to 0.2 μL are used, dilution of the initial suspension is not recommended. After inoculation, the plates are left at room temperature until the inoculation spots are completely absorbed into the agar (no more than 30 minutes). Incubate at 35 ± 2°C for 16 to 20 hours. The MIC value corresponds to the concentration in which a growth reduction of at least 80% is obtained, as compared to the control. The method should be conducted at least in duplicate. Although ADM remains the reference method for FOS MIC evaluation, it is not used routinely in diagnostic practice due to its labor-intensity and high time requirement (16-20 h) (Croughs et al., 2022). Alternative and faster methods, as gradient and disk diffusion test, or routinely used automated systems, as Vitek2, resulted unreliable due to their poor ability in detecting FOS^R^ isolates, with high error rates (van den Bijllaardt et al., 2018; Croughs et al., 2022). According to EUCAST guidelines, the disk diffusion test is intended only in investigating FOS profiles among *E. coli* strains, using 200 μg FOS disk and in presence of 50 μg of G6P.

### Commercial AD fosfomycin panel

A time-saving and ready-to-use alternative is represented by the commercial AD fosfomycin panel, commercialized in 2019 by Liofilchem S.r.l. (Roseto degli Abruzzi, Italy). The commercial AD fosfomycin panel allows FOS MIC evaluation and is composed of 12 wells filled with agar medium + 25 mg/L G6P and different concentrations of FOS (0.25–256 mg/L). The manufacturer’s guidelines provide for each isolates the preparation of a 0.5 McFarland bacterial suspension, consecutively diluted 1:10 in sterile saline solution. Each well is dispensed on agar surface with 2 μL (approximately 10^4^ CFU/spot) of the diluted bacterial suspension. The incubation step requires 35 ± 2°C for 16-20 hours in ambient air. FOS MIC is recorded as the lowest concentration of FOS that completely inhibit growth.

### Rapid fosfomycin/*E. coli* NP test

Nordmann and co-authors reported the description of a rapid test for FOS susceptibility profiles in *E. coli* (Nordmann et al., 2019). The rapid test is based on the microbial ability to metabolize glucose, that induce a colorimetric change of a specific pH indicator (culture medium, 2.5% MHB-CA powder, 0.005% phenol red indicator, and 1% **d** (+)-glucose). The test consists in preparing two solutions, named NP solutions: one solution with 25 μg/ml G6P and 40 μg/ml FOS, and one without. For bacterial suspension, a 3.0 to 3.5 McFarland solution for each tested isolate is prepared in 5 ml of sterile NaCl (0.85%). A 96-well polystyrene microtest plate is filled with both NP solutions and the bacterial suspension is directly inoculated in the presence or absence of FOS. After an incubation of 1 h 30 min at 35 ± 2°C, color changes are visually detected. FOS-resistant *E. coli* triggers a color change from orange to yellow, while FOS-susceptible remains orange (Nordmann et al., 2019).

This methodology showed both high rate of sensitivity (100%) and specificity (98.7%). In details, among 22 FOS-resistant *E. coli* isolates tested, all showed a positive result to the test (Nordmann et al., 2019). Similarly, Mueller and co-authors revealed a 100% correlation between susceptibility and resistance strains after screening 1,225 clinical ESβL-producing *E. coli* (Mueller et al., 2019). The rapid fosfomycin/*E. coli* NP test has the potential to be used as a rapid and first-step screening of FOS-resistant *E. coli*, thanks to its good performance and rapidity. A more recent evaluation on the accuracy of this rapid method was conducted on 149 clinical *E. coli* isolates, showing high rate of sensitivity and specificity (94.2% and 98.75%, respectively) and highlighting the reliability of the technique (Yunus et al., 2021). Differently, Kansak and colleagues found similar rate of sensitivity and specificity (95.9% and 100%, respectively) but a Very major Error (VME) of 22.2%, limiting the possibility to use the rapid test instead of ADM (Kansak et al., 2021). Despite the potential offered, the use of the rapid fosfomycin/*E. coli* NP test is still limited due to its applicability on *E. coli* only, the difficult in the interpretation of the results and the inability to distinguish between chromosomal and plasmid-acquired resistance mechanisms (Nordmann et al., 2019).

### SuperFOS selective medium

The SuperFOS selective medium provide a first line screening for FOS resistant *Enterobacterales*.

The SuperFOS medium combines the differentiation features of the CHROMagar orientation medium with an optimal concentration of FOS (16 μg/ml) and G6P (25 μg/ml). To avoid any contamination by eventual Gram-positive organism and fungi, the SuperFOS medium is enriched with vancomycin (20 μg/ml) and amphotericin B (5 μg/ml).

This medium provides several advantages due to its ease in preparation, the low cost, and the excellence performance, with both sensitivity and specificity at 100%. Moreover, the medium allows a first step screening of both chromosomal and plasmid mediated FOS^R^ mechanisms among *Enterobacterales* from clinical specimens (Nordmann et al., 2022).

### Disk potentiation testing with PPF

The disk potentiation testing with sodium phosphonoformate (PPF) is an agar-based diffusion test requiring the presence of FOS, G6P and PPF. PPF, commercially named Foscarnet, is an anti-viral compound used primarily in the treatment of CMV infections with inhibitory properties against FosA and FosC2 enzymes (Schreiber et al., 2009; Nakamura et al., 2014). PPF is able to bind FosA/FosC2 enzymes interacting with the residue MnII(+) and Thr9 that are present in the active site of FosA/FosC2-like enzymes, leading to a inhibitory effect and, thus, restoring the FOS susceptibility (Ito et al., 2017). The test requires MH agar plates with 25 mg/L G6P, 0.5 MacFarland solution of the isolate to investigate, two disk of FOS (50 μg) and PPF (1 mg). The cutoff is set to a 5 mm enlargement in the inhibition zone of FOS+PPF disk compared with the FOS disk alone (Nakamura et al., 2014). This agar-based method shows 100% sensitivity and specificity, and successfully detects the producing of enzymes FosA/A2 (Rigsby et al., 2004), FosA3, FosA4, FosA6 (Loras et al., 2021), FosA7 (Mattioni Marchetti et al., 2023), FosA8 (Biggel et al., 2021), FosA10 (Mattioni Marchetti et al., 2023), FosC2 (Nakamura et al., 2014), and FosL1 (Kieffer et al., 2020). However, the PPF test has been validated for *E. coli* strains only.

### Carbon source growth test

The carbon source growth test evaluates the ability of a bacterial strain to grow with G3P or G6P as the sole source of carbon. The inability to grow in presence of G3P and/or G6P is the result of a functional deficiency of the transporters GlpT and UhpT, respectively (Huang et al., 2021). This method requires the inoculation of the bacterial isolate on a M9 minimal medium agar supplemented with G3P or G6P at 0.2% (w/v) (Sorlozano-Puerto et al., 2020). After an incubation phase at 36°C for 48 h, the poor or total absence of growth is associated to an impairment in the transporter’s activity (Sorlozano-Puerto et al., 2020). The limitation of this growth test is mainly represented by the time required to perform it (72 h for results) and restricted results only on direct impairment of GlpT and UhpT activity.

## Limitations

This review presents several limitations. Few studies evaluate the prevalence of amino acidic mutations in proteins involved in FOS influx and their possible effect in FOS MIC increase (Kim et al., 1996; Takahata et al, 2010; Li et al., 2015). Whereby, the knowledge on specific mutations affecting FOS influx is not clear and incomplete.

Considering plasmid-mediated mechanisms for FOS^R^, the update global epidemiology of *fosA/fosC2/fosL1-2* gene is not completely and clearly monitored, mainly due to the lack of national surveillance plan, of fast methodology for the investigation of FOS^R^ profiles and the lack of general interest. Moreover, the characterization of *fosA*-like gene variants is so far only through molecular investigations and/or WGS. These point together, explain the difficulty to draw the updated epidemiology of FosA/C2/L1-2 enzymes and to clearly specific mutation decreasing FOS MICs.

Additionally, this review describes the FOS^R^ mechanisms that has been investigated and reported in literature among *Enterobacterales* only, while does not consider other relevant FOS^R^ sources, as *S. aureus* and *Enterococcus faecium*.

## Further perspective

FOS is still a valid option against MDR *Enterobacterales*, but this molecule is not always monitored routinely in clinical practice or in surveillance plans and, thus, the resistance mechanisms involved are not further investigated. In a *scenario* of increasing FOS^R^, time-saving and user-friendly methods for detecting such resistance profiles turn out to be fundamental. Implementation of faster testing would allow to conduct wide surveillance studies and to monitor FOS in clinical routine.

Time-saving methodologies aforementioned are validated for *E. coli* only. Therefore, the validation of these methods to further species would extend the pool of strains that can be tested, providing a more in-depth knowledge about FOS^R^ epidemiology. Moreover, a faster detection of FOS resistant bacteria and thus a further molecular characterization, could provide more information even on rarely reported FosA-like enzymes, such as FosC2, FosA4, FosA8 and FosA9, and could supply a more update epidemiology on *fosA/C2/L1-2* genes spread.

## Conclusion

Even though FOS is an old antimicrobial drug, it has unique and favorable features that lead in the last 20 years it to be considered as an additional resource in the treatment of MDR microorganisms’ infections (Michalopoulos et al., 2011). This review described the different mechanisms, identified so far, leading to FOS MIC increase among *Enterobacterales* genus. The FOS influx inside bacterial cell, that is regulated by different transporters and associated regulators, has also been described. Impairment in FOS transporters GlpT and UhpT is the most common mechanisms leading to the increase in FOS MICs, reported both *in vitro* and *in vivo* (Nilsson et al., 2003). The scientific community identified specific hotspot mutations in GlpT associated to a FOS resistance at high levels (FOS MICs > 128 μg/mL), such as W28del and Pro212Leu in *E. coli*, and as Arg206Lys and Ile293Phe in *K. pneumoniae* (Lu et al., 2016; Sorlozano-Puerto et al., 2020; Mattioni Marchetti et al., 2023). Compared with mutation frequency in GlpT and UhpT, modification in the target MurA were uncommon *in vivo* and no reports identified mutations in the active site (Cys115) in clinical isolates. In clinical *E. coli* strains the mutations Asp369Asn and Leu370Ile in MurA can likely develop FOS resistance profiles with MICs up to 512 mg/ml, while in clinical *K. pneumoniae* isolates the modifications Asp260Tyr and Thr307Lys has been associated to FOS MICs = 128 μg/mL (Takahata et al, 2010; Lu et al., 2016). The study of specific mutations in proteins involved in FOS influx and their eventual effect on FOS MICs is not in deep investigated and required further investigations.

Regarding acquired FOS^R^ mechanisms, in the last twelve years there has been a global diffusion of metallo-enzymes, named FosA-like, FosC2 and FosL1-L2 (Zurfluh et al., 2020). The Chinese clinical and veterinary environments show the highest frequency of FosA/C2 enzymes but, recently, many other countries as Brazil, Japan, Spain, and USA have reported such enzymes as well (Wachino et al., 2010; Jiang et al., 2017; Loras et al., 2021; Ewbank et al., 2022; Turcotte et al., 2022). To date, 11 variants of FosA enzymes has been identified, contributing to FOS resistance at different extents. In the global scenario, *fosA3* is the predominant type and it is widely reported in humans and veterinary settings. The wide and fast diffusion of *fosA3* has been facilitated by the combination of IS*26*-mediated transposons with epidemic broad-host-range plasmids as IncFII plasmids. The versatility of these *fosA3*-harboring plasmids has allowed the acquisition of *fosA3* genes in several clinically important ST such as *E. coli* ST10, *E. coli* ST69, *E. coli* ST131, *K. pneumoniae* ST11 and *S. enterica* ST32 (Xiang et al., 2015; Falagas et al., 2019; Seok et al., 2020). FosA3 is commonly co-expressed with other ESβLs, as CTX-M-65, and even with carbapenemases as KPCs, NDMs and VIMs (Villa et al., 2015; Xie et al., 2016; Jiang et al., 2017; Tang et al., 2020). Worryingly, the co-occurrence of *fosA3* + *mcr*-type genes in carbapenemases-producing *Enterobacterales* has been already described in the literature (Zhao et al., 2018; Peng et al., 2019; Tian et al., 2020).

Originated from *K. pneumoniae* chromosome, FosA5 and FosA6 can be considered among the most frequent metallo-enzyme leading to FOS^R^. However, their epidemiology has not been widely investigated in strains other than *K. pneumoniae* and the few reported cases are confined to countries as China and Spain (Guo et al., 2016; Wang et al., 2019). The diffusion of both *fosA5* and *fosA6* in *E. coli* is linked to IS*10* flaking cassettes (Xu et al., 2011; Dhanani et al., 2015).

Since the discover in 2015, FosA7 has rapidly spread among *Enterobacterales*, with high predominance among *Salmonella* spp. So far, nine alleles of *fosA7* have been described and deposited in the GenBank. *FosA7*-like genes are strictly located on *Salmonella* spp. chromosome, except for *fosA7.5* and *fosA7.9* that are associated to *E. coli* and *C. freundii* chromosome, respectively. The current spread of *fosA7*-like genes includes countries as Canada and China (Dhanani et al., 2015; Pan et al., 2021).

Concurrence of impairing mutations in FOS influx and acquisition of *fosA/C2/L1-2* together with ESβLs and carbapenemases genes, is worrying and could strongly affect the use of FOS in severe infections treatment.

ADM is the reference methods for FOS MICs evaluation and the few rapid methods available have been validated for *E. coli* only or are prone to error. The increase of surveillance plans and the implementation of new rapid approaches for the detection of FOS^R^ *Enterobacterales*, would favorite a better and in-depth knowledge on the prevalence of FOS^R^ mechanisms. Moreover, a clearer information on such mechanisms and their dissemination results of priority importance to halt eventual FOS^R^ dissemination and to optimize therapeutic strategies.

## Author contributions

VM, IB, and JH played an important role in searching the relevant literature, writing and correcting the manuscript. All authors contributed to the article and approved the submitted version.
